# ABC transporter mis-splicing associated with resistance to Bt toxin Cry2Ab in laboratory- and field-selected pink bollworm

**DOI:** 10.1038/s41598-018-31840-5

**Published:** 2018-09-10

**Authors:** Lolita G. Mathew, Jeyakumar Ponnuraj, Bheemanna Mallappa, Lingutla R. Chowdary, Jianwei Zhang, Wee Tek Tay, Thomas K. Walsh, Karl H. J. Gordon, David G. Heckel, Sharon Downes, Yves Carrière, Xianchun Li, Bruce E. Tabashnik, Jeffrey A. Fabrick

**Affiliations:** 1 U.S. Department of Agriculture (USDA), Agricultural Research Service (ARS), U.S. Arid Land Agricultural Research Center, Maricopa, AZ 85138 USA; 2 Indian Council of Agricultural Research (ICAR), Indian Institute of Rice Research (IIRR), Rajendra Nagar, Hyderabad, 500 030 India; 3 Pesticide Residue and Food Quality Analysis Laboratory, University of Agricultural Sciences, Raichur, Karnataka 584 104 India; 4 Agriculture Research Station, Acharya N.G. Ranga Agricultural University, Darsi, Andhra Pradesh 523 247 India; 5 Arizona Genomics Institute, University of Arizona, Tucson, AZ 85721 USA; 6 Commonwealth Scientific and Industrial Research Organization (CSIRO), Black Mountain Laboratories, Acton, ACT 2601 Australia; 7 Department of Entomology, Max Planck Institute for Chemical Ecology, 07745 Jena, Germany; 8 Commonwealth Scientific and Industrial Research Organization (CSIRO), Myall Vale Laboratories, Narrabri, NSW 2390 Australia; 9 Department of Entomology, University of Arizona, Tucson, AZ 85721 USA

## Abstract

Evolution of pest resistance threatens the benefits of genetically engineered crops that produce *Bacillus thuringiensis* (Bt) insecticidal proteins. Strategies intended to delay pest resistance are most effective when implemented proactively. Accordingly, researchers have selected for and analyzed resistance to Bt toxins in many laboratory strains of pests before resistance evolves in the field, but the utility of this approach depends on the largely untested assumption that laboratory- and field-selected resistance to Bt toxins are similar. Here we compared the genetic basis of resistance to Bt toxin Cry2Ab, which is widely deployed in transgenic crops, between laboratory- and field-selected populations of the pink bollworm (*Pectinophora gossypiella*), a global pest of cotton. We discovered that resistance to Cry2Ab is associated with mutations disrupting the same ATP-binding cassette transporter gene (*PgABCA2*) in a laboratory-selected strain from Arizona, USA, and in field-selected populations from India. The most common mutation, loss of exon 6 caused by alternative splicing, occurred in resistant larvae from both locations. Together with previous data, the results imply that mutations in the same gene confer Bt resistance in laboratory- and field-selected strains and suggest that focusing on *ABCA2* genes may help to accelerate progress in monitoring and managing resistance to Cry2Ab.

## Introduction

The insecticidal proteins produced by *Bacillus thuringiensis* (Bt) kill some devastating insect pests, but cause little or no harm to humans and most other non-target organisms^1,2^. Farmers have used Bt toxins in insecticidal sprays for more than 70 years and in transgenic crops since 1996^3–5^. In 2017, genetically engineered corn, cotton and soy producing Bt toxins were planted by millions of farmers on more than 100 million hectares worldwide^5^. The benefits of these Bt crops include pest suppression, reduced insecticide use, enhanced biological control, and increased farmer profits^2,6–11^. However, increasingly rapid evolution of pest resistance to Bt toxins has reduced these benefits^12^.

Strategies designed to delay pest resistance to Bt crops are most likely to succeed if they are implemented proactively, before resistance is widespread^13,14^. To provide the information needed to bolster the proactive development of such strategies, researchers have selected for and analyzed resistance to Bt toxins in many laboratory strains of pests^4,15^. The utility of this approach depends on the largely untested assumption that laboratory- and field-selected resistance are similar^16^.

Here we tested the hypothesis that the molecular genetic basis of resistance to Bt toxin Cry2Ab is similar in laboratory- and field-selected populations of one of the world’s most destructive pests of cotton, the pink bollworm (*Pectinophora gossypiella*)^17,18^. Understanding the genetic basis of insect resistance to Cry2Ab is increasingly important for several reasons. While each of the first Bt crops produced a single toxin from the Cry1 family (e.g., Cry1Ac), most Bt crops grown now produce Cry2Ab in combination with one or more Cry1 toxins^19^. Whereas at least eight major lepidopteran pests have evolved resistance to Cry1 toxins in the field, field-evolved resistance to Cry2Ab has been reported only for pink bollworm in India and *Helicoverpa zea* in the United States^12,20–23^. In India, over 7 million farmers cultivated the largest area of Bt cotton (10.8 million ha) of any nation in 2016^5^ and pink bollworm resistance to Cry1Ac and Cry2Ab produced by dual-toxin Bt cotton has caused severe economic losses^20,21,24–26^. Moreover, data reported previously on the genetic basis of resistance are extensive for Cry1 toxins^12,15^, but are relatively scarce and limited to laboratory-selected strains for Cry2Ab^27–29^. Resistance to Cry2Ab has been linked with mutations in an ATP-binding cassette transporter gene (*ABCA2*) in lab-selected strains of *Helicoverpa armigera* and *Helicoverpa punctigera* from Australia^28^, but we are not aware of any previous reports of the molecular genetic basis of field-evolved resistance to Cry2Ab.

Here we discovered that pink bollworm resistance to Cry2Ab is associated with mutations disrupting *ABCA2* in a laboratory-selected strain from Arizona, USA, and in field-selected populations from India. Aberrant splicing of pre-mRNA was prevalent in the mutations affecting this gene in resistant pink bollworm from Arizona and India. Although one mutation was shared by some resistant larvae from both locations, the others differed between locations and were strikingly diverse in India. The results suggest that focusing on *ABCA2* may help to accelerate progress in monitoring and managing field-evolved resistance to Cry2Ab.

## Results

### ABCA2 in Susceptible and Lab-Selected Resistant Pink Bollworm from Arizona

**Figure 1:**
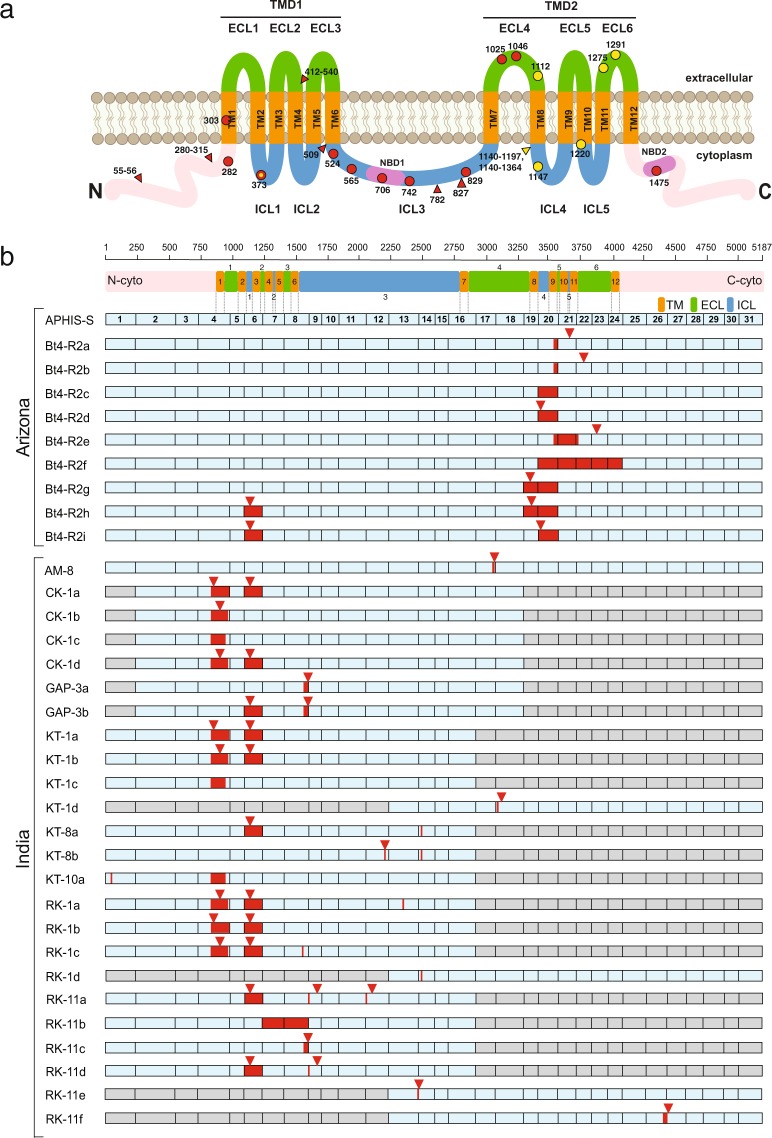
Mutations affecting PgABCA2 protein in Cry2Ab-resistant pink bollworm from Arizona and India. (**a**) The predicted PgABCA2 protein includes amino (N) and carboxyl (C) termini (pink), two transmembrane domains (TMD1 and TMD2), each consisting of 6 transmembrane regions (TM; orange), three extracellular loops (ECL; green), two intracellular loops (ICL; blue), and two nucleotide-binding domains (NBD; purple). Mutations affecting transcripts of resistant pink bollworm: Circles show premature stop codons from India (red), Arizona (yellow), or both (red and yellow). Triangles show in-frame indels from India (red) or Arizona (yellow). Numbers indicate the affected amino acids. (**b**) Full-length and partial *PgABCA2* cDNAs were obtained by direct PCR sequencing, DNA sequencing of cDNA clones, and/or PacBio^®^ DNA sequencing from susceptible (APHIS-S) and resistant, laboratory-selected (Bt4-R2) pink bollworm from Arizona, USA and India field-selected resistant populations (AM, CK, GAP, KT, and RK). The linear schematic (top) shows the predicted translated domain structure of the 5,187-bp full-length *PgABCA2* coding sequence. The predicted protein includes amino- and carboxyl-termini (pink), transmembrane regions TM1-TM12 (orange), intracellular loops ICL1-ICL5 (blue), and extracellular loops ECL1-ECL6 (green). The domain structure connected to the exons that encode the respective domains is shown by dotted gray lines. Each predicted domain is numbered, with ECLs on top and ICLs numbered on bottom of protein schematic. Putative exons 1–31 are numbered, with grey exons indicating regions determined by direct PCR sequencing. Exons colored in light blue were further verified by sequencing cDNA clones (either by Sanger or PacBio^®^ sequencing). Red bars indicate disruption sites within the full-length coding sequence and the red triangles indicate the location of premature stop codons shown to scale based on the linear schematic of the translated domain structure. Unique cDNA variants are indicated as a, b, c, etc.

The full-length consensus *PgABCA2* cDNA (GenBank accession no. MG637361) from two Arizona Cry2Ab-susceptible strains, APHIS-S and AZP-R, was identical and had 5,187 bp encoding a predicted protein of 1,729 aa (Supplementary Fig. S1). Similar to other ABC transporters in subfamily A, the proposed structure of PgABCA2 includes two transmembrane domains (TMD1 and TMD2) and two nucleotide-binding domains (NBD1 and NBD2) (Fig. 1a and Supplementary Fig. S1). Each transmembrane domain has six transmembrane helices and both nucleotide-binding domains have a catalytic core domain consisting of Walker A and B motifs and a structurally diverse α-helical domain containing the ABC transporter signature motif (also called Walker C motif) in the cytoplasm.

We initially identified four transcript variants (Bt4-R2a to d) with mutations in *PgABCA2* from cDNA sequencing of a pool of 10 larvae from the laboratory-selected Cry2Ab-resistant strain Bt4-R2 (Fig. 1, Supplementary Fig. S2, and Table 1). Each of these mutations affects exon 20: either a frameshift mutation introducing a premature stop codon truncating the PgABCA2 protein (Bt4-R2a, b, and d), or an in-frame deletion of exon 20 disrupting intracellular loop 4, transmembrane helix 9, and extracellular loop 5 (Bt4-R2c) (Fig. 1).

**Table 1:** Unique *PgABCA2* transcript variants and cDNA mutations in Cry2Ab-resistant pink bollworm from Arizona.

Variant^a^	Source^b^	cDNA mutation^c^	Codon^d^	Exon^e^	Mis-splicing^f^	Type^g^	Effect^h^
Bt4-R2a	Bt4-R2	c.3556_3588delins^i^	1186	20	complete intron 20 retention	fs	stop at 1220
Bt4-R2b	Bt4-R2	c.3556_3589_delins^i^	1186	20	alternative 5′ splice site in intron 20	fs	stop at 1275
Bt4-R2c	Bt4-R2	c.3419_3589delinsTAA	1140–1197	20	alternative 5′ splice site in intron 19 and exon 20 skip	InF	In-frame indel
Bt4-R2d	Bt4-R2	c.3419_3590del	1140	20	exon 20 skip	fs	stop at 1147
Bt4-R2e	H-5	c.3556_3734delins^i^	1186	20–22	alternative 5′ splice site in intron 20, exon 21 skip, and alternative 3′ splice site in exon 22	fs	stop at 1291
Bt4-R2f	J-3	c.3419_4093del	1140–1364	20–24	exon 20–23 skip and alternative 3′ splice site in exon 24	InF	In-frame indel
Bt4-R2g	H-5	c.3313_3589del	1105	19–20	exon 19–20 skip	fs	stop at 1112
Bt4-R2h	H-1	c.1090_1234del	364	6	exon 6 skip	fs	stop at 373
		c.3313_3589del	1105	19–20	exon 19–20 skip	fs	stop at 1112
Bt4-R2i	G-2	c.1090_1234del	364	6	exon 6 skip	fs	stop at 373
		c.3419_3590del	1140	20	exon 20 skip	fs	stop at 1147

^a^Transcript variant names include the strain (Bt4-R2) followed by a lower-case letter designating the specific transcript variant. Only unique transcript variants are shown. Bt4-R2a-d transcript variants are from cloning and Sanger sequencing. Bt4-R2e-i transcript variants are from PacBio® targeted sequencing of *r*_*A1*_*r*_*A1*_ backcross survivors from genetic linkage crosses. ^b^Bt4-R2a-d were identified from a pool of 10 larvae from Bt4-R2; for Bt4-R2e-i, the backcross family name is listed for each of the individual larvae analyzed that had survived exposure to Cry2Ab in the linkage analysis (Supplementary Table S2). ^c^cDNA mutation nomenclature is based on the recommendations by the Human Genome Variation Society (http://www.hgvs.org/). ^d^Codon number beginning from the initiation codon and indicating disrupted position within the coding sequence. ^e^Disrupted exon within the coding sequence. ^f^Event causing mis-spliced transcripts (complete intron retention, alternative 5′- or 3′-splice sites, and/or exon skip). ^g^Type of mutation (fs, frame shift; InF, In-frame mutation). ^h^Result and codon position of mutation in the coding sequence (introduction of premature stop codon or In-frame indel). ^i^Actual sequences corresponding to insertions not shown here due to large number of nucleotide bases.

**Figure 2:**
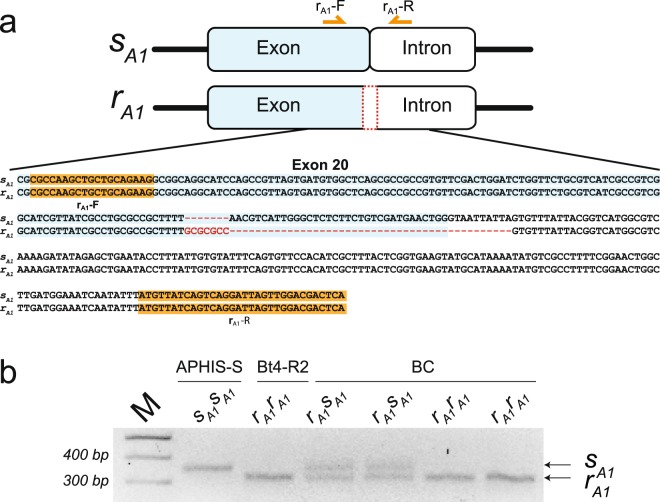
The *r*_*A1*_ mutation at the junction of exon and intron 20 in *PgABCA2*. (**a**) gDNA sequence for exon and intron 20 of the wild-type *PgABCA2* allele (*s*_*A1*_) in the susceptible APHIS-S strain and the mutant *r*_*A1*_ allele from the Cry2Ab-resistant Bt4-R2 strain. The *r*_*A1*_ indel mutation has a 7-bp insertion (red letters) and 44-bp deletion (red dashes) in exon 20 (blue) and spanning the 5′ splice junction with intron 20 (not highlighted). The allele-specific primers r_A1_-F and r_A1_-R match the sequences highlighted in orange. (**b**) Allele-specific PCR using the primers in (**a**) yielded a 343-bp fragment from APHIS-S (*s*_*A1*_*s*_*A1*_), a 305-bp fragment from Bt4-R2 (*r*_*A1*_*r*_*A1*_), and both fragments in the offspring from crosses between the two strains (*r*_*A1*_*s*_*A1*_). Unprocessed image of agarose gel is shown in Supplementary Fig. S13.

To test for differences between the susceptible APHIS-S strain and the resistant Bt4-R2 strain in genomic DNA (gDNA) near exon 20 of *PgABCA2*, we PCR amplified, cloned, and sequenced fragments from exon 18 to exon 21. Compared with APHIS-S, the gDNA from Bt4-R2 contained an indel mutation expected to disrupt the 3′ splice junction of exon 20 (Fig. 2a and Supplementary Fig. S2). This splice-site mutation includes a 7-bp insertion (GCGCGCC), followed by a 44-bp deletion that spans the exon-intron junction. We name this mutation “*r*_*A1*_” and refer to the allele containing this mutation as the *r*_*A1*_ allele. Because the *r*_*A1*_ mutation spans the exon-intron splice junction, the Bt4-R2a-d cDNA variants probably result from mis-splicing of intron 20. Further examination of nearby gDNA revealed that, relative to APHIS-S, Bt4-R2 also has a 1,129-bp insertion in intron 18 (Supplementary Fig. S2).

To distinguish readily between the *r*_*A1*_ resistance allele from Bt4-R2 and the susceptible “*s*_*A1*_” allele from APHIS-S, we developed allele-specific PCR using primers that flank the *r*_*A1*_ mutation (Fig. 2 and Supplementary Table S1). These primers yielded a 343-bp fragment from APHIS-S individuals (*s*_*A1*_*s*_*A1*_), a 305-bp fragment from Bt4-R2 (*r*_*A1*_*r*_*A1*_), and both fragments in the Bt4-R2 X APHIS-S (*r*_*A1*_*s*_*A1*_) F_1_ offspring from biphasic linkage crosses detailed below (Fig. 2b).

### Genetic Linkage between *PgABCA2* and Resistance to Cry2Ab

**Figure 3:**
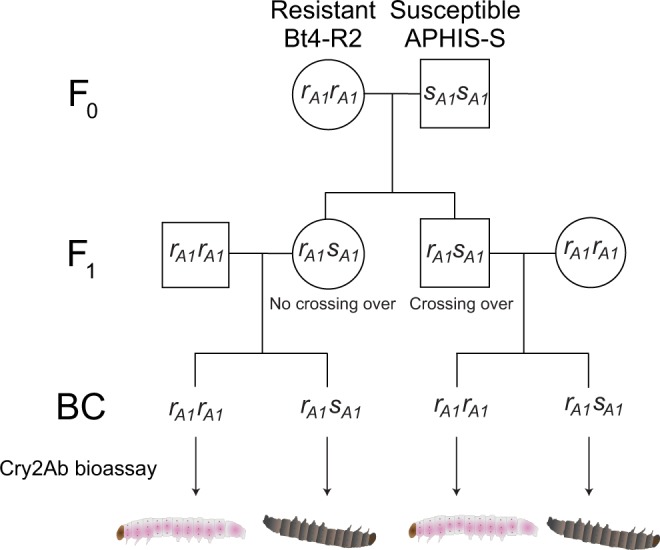
Linkage analysis. Biphasic genetic linkage analysis was initiated with single-pair reciprocal crosses between F_0_ adults, with either a resistant Bt4-R2 female and a susceptible APHIS-S male (shown here) or a susceptible APHIS-S female and a resistant Bt4-R2 male. The resulting F_1_ progeny were backcrossed in single pairs with the resistant Bt4-R2 strain. Backcross (BC) progeny were tested either on diet containing 1 microgram Cry2Ab per mL diet or control diet. Survivors from 10 reciprocal backcross families (five F_1_ ♀ X Bt4-R2 ♂ and five Bt4-R2 ♀ X F_1_ ♂) were weighed and genotyped with allele-specific PCR.

We tested for genetic linkage between *PgABCA2* and resistance to Cry2Ab using backcross progeny obtained from single-pair crosses between Bt4-R2 (*r*_*A1*_*r*_*A1*_) and F_1_ (*r*_*A1*_*s*_*A1*_, from Bt4-R2 X APHIS-S) (Fig. 3 and Supplementary Table S2). From an initial set of 42 backcross families, we tested larval survival in bioassays with 1 microgram Cry2Ab per ml diet for 20 backcross families that each produced at least 10 progeny (mean = 28 neonates tested per family) (Supplementary Fig. S4). We used the allele-specific PCR described above to determine the *PgABCA2* genotype of larvae.

In the 10 backcross families where the father was from Bt4-R2, the survivors on Cry2Ab-treated diet consisted of 125 *r*_*A1*_*r*_*A1*_ and 9 *r*_*A1*_*s*_*A1*_, which differs significantly from the ratio of 1:1 expected if resistance is not linked with *PgABCA2* (Fisher’s exact test, P < 10^−14^) (Supplementary Table S2). Because crossing over in Lepidoptera occurs only in males, these results from backcross families with *r*_*A1*_*r*_*A1*_ fathers indicate linkage between resistance and the chromosome carrying *PgABCA2*. In the other 10 backcross families, where the father was an F_1_, the survivors on Cry2Ab-treated diet consisted of 113 *r*_*A1*_*r*_*A1*_ and 7 *r*_*A1*_*s*_*A1*_, which also differs significantly from the expected 1:1 ratio if resistance is not linked with *PgABCA2* (Fisher’s exact test, P < 10^−14^) (Supplementary Table S2). Because crossing over occurs in males, the results from these 10 backcross families with *r*_*A1*_*s*_*A1*_ fathers indicate tight linkage between resistance and *PgABCA2*. Furthermore, in the backcross progeny that survived exposure to Cry2Ab, the proportion of individuals with genotype *r*_*A1*_*r*_*A1*_ was not higher for families with *r*_*A1*_*r*_*A1*_ fathers (0.93) where crossing over was not a factor, than for families with *r*_*A1*_*s*_*A1*_ fathers (0.94) where crossing over could occur, which implies tight linkage between resistance and *PgABCA2*. By contrast with the results described above, larvae from backcross families reared on untreated diet as controls consisted of 15 *r*_*A1*_*r*_*A1*_ and 12 *r*_*A1*_*s*_*A1*_, which does not differ significantly from the expected 1:1 ratio (Fisher’s exact test, P = 0.79).

For survivors on diet treated with Cry2Ab in the linkage analysis, mean larval weight (mg) was significantly lower for *r*_*A1*_*s*_*A1*_ (19, SE = 2.6) than *r*_*A1*_*r*_*A1*_ (27, SE = 0.5) (t-test, t = 3.8, df = 250, P = 0.0002) (Supplementary Fig. S4). However, larval weight on untreated diet was similar for *r*_*A1*_*s*_*A1*_ (26, SE = 1.9) and *r*_*A1*_*r*_*A1*_ (26, SE = 0.5, SE = 2.8) (t-test, t = 0.14, df = 41, P = 0.89). In addition, larval weight was lower on treated diet versus untreated diet for *r*_*A1*_*s*_*A1*_ (t-test, t = 2.2, df = 36, P = 0.03), but not for *r*_*A1*_*r*_*A1*_. Thus, in terms of larval weight, resistance to Cry2Ab was incomplete in the relatively rare *r*_*A1*_*s*_*A1*_ survivors, compared with complete resistance in the more abundant *r*_*A1*_*r*_*A1*_ survivors. The low level of resistance in *r*_*A1*_*s*_*A1*_ could reflect inheritance of resistance linked with *PgABCA2* that was not completely recessive, a contribution to resistance from one or more mutations other than *r*_*A1*_, or both.

### Single-molecule Long-read Sequencing of *PgABCA2* cDNA

To check the accuracy of the genotypes assigned by PCR and to determine if Bt4-R2 has mutations in *PgABCA2* other than the *r*_*A1*_ splice-site mutation, we used single-molecule, real-time sequencing to analyze barcoded *PgABCA2 cDNA* from 22 larvae: seven from APHIS-S as controls, and 15 backcross survivors from the linkage analysis (Fig. 3) that had been genotyped with allele-specific PCR (eight *r*_*A1*_*r*_*A1*_ and seven *r*_*A1*_*s*_*A1*_). We obtained a total of 369,672 reads, which yielded consensus sequences for 18 of the 22 larvae (Supplementary Table S3). For the other four larvae (two APHIS-S and two *r*_*A1*_*r*_*A1*_), the coverage was not deep enough to give reliable consensus sequences. For 16 of the 18 larvae with consensus sequences, the cDNA sequences confirmed the results from PCR in terms of the presence or absence of the *r*_*A1*_ mutation. As expected, the five APHIS-S larvae had only intact cDNA (*s*_*A1*_*s*_*A1*_), all of the transcripts from the six larvae genotyped by PCR as *r*_*A1*_*r*_*A1*_ had mutations affecting exon 20, and four of the six larvae genotyped by PCR as *r*_*A1*_*s*_*A1*_ each had at least one transcript with the *r*_*A1*_ mutation and one without it (Table 1 and Supplementary Table S3). For the other two larvae genotyped by PCR as *r*_*A1*_*s*_*A1*_ (*r*_*A1*_*s*_*A1*_-6 and *r*_*A1*_*s*_*A1*_-7), targeted sequencing yielded consensus reads with the intact sequence (*s*_*A1*_) in the region of the *r*_*A1*_ mutation, but not the expected *r*_*A1*_ mutation (Supplementary Table S3). However, subsequent cDNA cloning and Sanger sequencing confirmed the presence of the *r*_*A1*_ mutation. Thus, both of these larvae had transcripts with and without the *r*_*A1*_ mutation, confirming the PCR genotyping.

The long-read sequencing also revealed five previously unidentified transcript variants (Bt4-R2e to i), bringing the total of different cDNA variants in Bt4-R2 that disrupt *PgABCA2* to nine, each having either one or two of the eight different cDNA mutations affecting exon 6 or one or more exons from 19 to 24 (Fig. 1 and Supplementary Fig. S2; Table 1 and Supplementary Table S3). Four of the 12 larval survivors from the backcross analyzed by targeting sequence shared a cDNA mutation (c.1090_1234del) that completely skips exon 6 (1,090–1,234 bp), yielding a frameshift and a premature stop codon at amino acid 373 (Fig. 1 and Supplementary Fig. S2; Table 1 and Supplementary Table S3). For one of these four larvae (*r*_*A1*_*s*_*A1*_-7), the transcript without the *r*_*A1*_ mutation lacked exon 6 and thus was not an intact, wild-type transcript. Therefore, when considering the entire *PgABCA2* cDNA sequence (rather than just the region harboring the *r*_*A1*_ mutation), this individual had only disrupted transcripts, which may explain its ability to survive when exposed to Cry2Ab.

Although we initially identified only 4 individuals (*r*_*A1*_*s*_*A1*_-1, *r*_*A1*_*s*_*A1*_-2, *r*_*A1*_*s*_*A1*_-6 and *r*_*A1*_*s*_*A1*_-7) with cDNA mutation that completely skip exon 6, we found 6 more individuals (two homozygous: *r*_*A1*_*r*_*A1*_-3, *r*_*A1*_*r*_*A1*_-5 and 4 heterozygous: *r*_*A1*_*s*_*A1*_-2, *r*_*A1*_*s*_*A1*_-3, *r*_*A1*_*s*_*A1*_-4 and *r*_*A1*_*s*_*A1*_-5) when we changed the conditions to include shorter subreads in the Long Amplicon Analysis (Supplementary Table S3). We cloned and Sanger sequenced the gDNA from three individuals (*r*_*A1*_*s*_*A1*_-3, *r*_*A1*_*s*_*A1*_-4 and *r*_*A1*_*s*_*A1*_-6) and found no changes in gDNA corresponding to the observed loss of exon 6 in cDNA (Supplementary Fig. S5).

Including the results from the initial sequencing of 10 pooled resistant larvae and next generation sequencing of 15 individual survivors from the linkage analysis (Table 1 and Supplementary Table S3), mis-splicing is implicated in each of the eight different cDNA mutations in Bt4-R2. Comparison of the Bt4-R2 genomic DNA sequence and the transcript variants at exon 20 revealed that the indel mutation at the splice junction disrupted the 5′ GT intron signature sequence and introduced several cryptic splice sites in the pre-mRNA (Table 1 and Supplementary Fig. S6). The indel mutation thus caused mis-splicing of the Bt4-R2 pre-mRNA and produced nine transcript variants with disruptions in exons 19–24 (Table 1 and Supplementary Fig. S6).

### Mutations in *PgABCA2* Associated with Field-Selected Resistance to Bt Cotton in India

**Figure 4:**
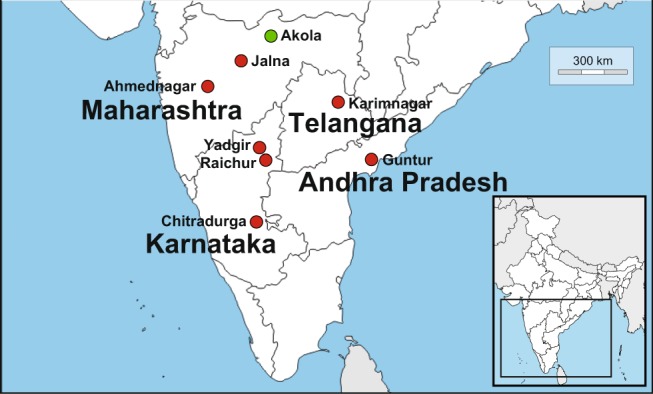
Pink bollworm sampling sites in India. After widespread resistance to Cry2Ab was reported in India, Cry2Ab-resistant larvae were collected in 2015 and 2016 as fourth instars from dual-toxin cotton producing Cry2Ab and Cry1Ac from seven districts of four states: Ahmednagar and Jalna, Maharashtra (AM and JM), Karimnagar, Telangana (KT), Guntur, Andhra Pradesh (GAP), and Chitradurga, Raichur and Yadgir, Karnataka (CK, RK and YK) (red). Several years before resistance to Cry2Ab was detected in India, Cry2Ab-susceptible larvae were collected in 2010 from non-Bt cotton in Akola, Maharashtra (AMH) (green).

To determine if mutations in *PgABCA2* are associated with field-selected resistance to Cry2Ab, we compared cDNA from field-collected pink bollworm larvae from India that were either susceptible or resistant to Cry2Ab. We collected the three susceptible larvae from a non-Bt cotton field in Akola, Maharashtra (AMH-1, AMH-2, and AMH-3) in 2010^30^, five years before resistance to Cry2Ab was detected in India (Fig. 4). During December 2015 and January 2016, after pink bollworm resistance to Cry2Ab was widespread in India^20,25^, we collected 11 larvae as fourth instars from fields of dual-toxin Bt cotton at seven sites in India (Fig. 4). We identified these larvae as resistant because immunoassays confirmed that the bolls from which they were collected produced both Cry2Ab and Cry1Ac (Supplementary Table S4). We obtained sufficient material for full-length cDNA sequencing for all three susceptible larvae and eight resistant larvae from five sites in India (AM-8, CK-1, GAP-3, KT-1, KT-8, KT-10, RK-1, and RK-11).

The consensus cDNA sequences of *PgABCA2* were identical for the three susceptible larvae from India and the susceptible APHIS-S strain from Arizona (Supplementary Fig. S7). Conversely, the eight resistant larvae from five sites in India yielded 19 distinct *PgABCA2* transcript variants, each severely disrupted by one to three mutations (Fig. 1 and Table 2). Collectively, the eight resistant larvae from India harbored 17 different cDNA mutations, and a total of 40 mutations including several that occurred in more than one larva (Table 2). The disruptive effects of these 40 *PgABCA2* cDNA mutations are expected throughout the encoded protein (Fig. 1).

**Table 2:** Unique *PgABCA2* transcript variants and cDNA mutations in Cry2Ab-resistant pink bollworm from India.

Larva^a^	Variant^b^	cDNA mutation^c^	Codon^d^	Exon^e^	Type^f^	Effect^g^
AM-8	AM-8a	c.3065_3069del	1022	17	fs	stop at 1025
CK-1	CK-1a	c.838_979del	280	4	fs	stop at 282
		c.1090_1234del	364	6	fs	stop at 373
	CK-1b	c.837_964del	280	4	fs	stop at 303
	CK-1c	c.837_944delins^h^	280–315	4	InF	In-frame indel
	CK-1d	c.837_964del	280	4	fs	stop at 303
		c.1090_1234del	364	6	fs	stop at 373
GAP-3	GAP-3a	c.1572_1618del	524	8	InF	stop at 524
	GAP-3b	c.1090_1234del	364	6	fs	stop at 373
		c.1572_1618del	524	8	InF	stop at 524
KT-1	KT-1a	c.838_979del	280	4	fs	stop at 282
		c.1090_1234del	364	6	fs	stop at 373
	KT-1b	c.837_964del	280	4	fs	stop at 303
		c.1090_1234del	364	6	fs	stop at 373
	KT-1c	c.837_944delins^h^	280–315	4	InF	In-frame indel
	KT-1d	c.3098_3101del	1033	18	fs	stop at 1046
KT-8	KT-8a	c.1090_1234del	364	6	fs	stop at 373
		c.2479_2481del	827	14	InF	In frame del
	KT-8b	c.2224 A > T	742	12	InF	stop at 742
		c.2479_2481del	827	14	InF	In frame del
KT-10	KT-10a	c.55_56delinsCA	55–56	1	InF	In-frame indel
		c.837_944delins^h^	280–315	4	InF	In-frame indel
RK-1	RK-1a	c.837_964del	280	4	fs	stop at 303
		c.1090_1234del	364	6	fs	stop at 373
		c.2344_2345delinsGG	782	13	InF	In-frame indel
	RK-1b	c.838_979del	280	4	fs	stop at 282
		c.1090_1234del	364	6	fs	stop at 373
	RK-1c	c.837_964del	280	4	fs	stop at 303
		c.1090_1234del	364	6	fs	stop at 373
		c.1526_1527delinsGC	509	8	InF	In-frame indel
	RK-1d	c.2479_2481del	827	14	InF	In-frame del
RK-11	RK-11a	c.1090_1234del	364	6	fs	stop at 373
		c.1622_1623ins^h^	541	9	fs	stop at 565
		c.2065_2066ins^h^	689	12	fs	stop at 706
	RK-11b	c.1235_1618del	412–540	7–8	InF	In-frame indel
	RK-11c	c.1572_1618del	524	8	InF	stop at 524
	RK-11d	c.1090_1234del	364	6	fs	stop at 373
		c.1622_1623ins^h^	541	9	fs	stop at 565
	RK-11e	c.2464_2465ins^h^	822	14	fs	stop at 829
	RK-11f	c.4389_4440del	1464	26–27	fs	stop at 1475

^a^cDNA was obtained and full-length PCR products and partial cloned cDNA fragments were sequenced from eight resistant pink bollworm larvae collected from Bollgard II® cotton bolls from India containing Cry2Ab + Cry1Ac. ^b^Transcript variants are named based on location (AM, CK, GAP, KT, or RK; Fig. 4), a number designating the specific individual within each site, and a lower-case letter designating the specific variant within each individual larva. ^c^cDNA nomenclature showing the nucleic acid sequence changes based on the recommendations by the Human Genome Variation Society (http://www.hgvs.org/). ^d^Codon number beginning from the initiation codon and indicating disrupted position within the coding sequence. ^e^Disrupted exon within the coding sequence. ^f^Type of mutation (fs, frame shift; InF, In-frame mutation). ^g^Result and codon position of mutation in the coding sequence (Introduction of premature stop codon; In-frame indel; In-frame deletion). ^h^Sequences corresponding to insertions not shown here due to large number of nucleotide bases.

The most common cDNA mutation in the resistant larvae from India was loss of exon 6, exactly the same mutation as in the four larvae from Bt4-R2 described above (Table 1 and Fig. 1). This mutation occurred in 75% (6 of 8) of the resistant larvae from India representing all four states studied here (Table 2). In 18 clones from four resistant larvae from India with this cDNA mutation, we found no changes in the corresponding gDNA at or near the exon/intron boundaries of exon 6 (Supplementary Figs S8–S11 and Table 3), which implicates alternative splicing. Thus, in a lab-selected strain from Arizona and field-selected populations from India, pink bollworm resistance to Cry2Ab is associated with mis-splicing that omits exon 6 of *PgABCA2*.

**Table 3:** Mis-splicing of *PgABCA2* in Cry2Ab-resistant pink bollworm from India.

Larva^a^	cDNA mutation^b^	Exon^c^	Type^d^	Splicing affected^e^	Mis-splicing^f^
AM-8	c.3065_3069del	17	fs	no	none
CK-1	c.838_979del	4	fs	yes	alternative 5′ splice site in exon 4
	c.837_964del	4	fs	yes	alternative 5′ splice site in exon 4 and alternative 3′ splice site within insertion in exon 4
	c.837_944delins	4	InF	yes	alternative 5′ and 3′ splice sites in exon 4
	c.1090_1234del	6	fs	yes	exon 6 skip
GAP-3	c.1090_1234del	6	fs	yes	exon 6 skip
	c.1572_1618del	8	InF	yes	alternative 5′ splice site in exon 8
KT-1	c.1090_1234del	6	fs	yes	exon 6 skip
RK-1	c.837_964del	4	fs	yes	alternative 5′ and 3′ splice sites in exon 4
	c.838_979del	4	fs	yes	alternative 5′ splice site in exon 4
	c.1090_1234del	6	fs	yes	exon 6 skip

^a^gDNA was extracted from five resistant larvae from India and PCR amplicons corresponding to cDNA with mutations were cloned and sequenced by Sanger sequencing. ^b^cDNA nomenclature based on the recommendations by the Human Genome Variation Society (http://www.hgvs.org/). ^c^Disrupted exon in the coding sequence. ^d^Type of mutation: fs, frame shift; InF, In-frame mutation. ^e^Determined by direct comparison of cDNA and gDNA sequences (see Supplementary Figs. S8–12). ^f^Event causing mis-spliced transcripts (alternative 5′- or 3′-splice sites and/or exon skip).

By contrast, we did not detect the *r*_*A1*_ splice-site mutation from Arizona in any of the 11 resistant larvae from India. We did not find any mutations in exon 20 in full-length *PgABCA2* cDNA sequences from the eight resistant larvae from India noted above. In addition, the *r*_*A1*_ mutation was not detected with allele-specific PCR in four of the resistant larvae mentioned above (AM-8, CK-1, KT-1, and RK-1) or in three additional resistant larvae from India that were not fully sequenced (GAP-2, JM-9, and YK-1).

Including 4 of the 19 distinct *PgABCA2* cDNA sequences that occurred in larvae from two to three sites each, we found a total of 24 mutant transcripts (Table 2). The four transcript variants detected at more than one site are: CK-1a, KT-1a, and RK-1b with stop codons at amino acids 282 and 373; CK-1d and KT-1c with stop codons at amino acids 303 and 373; GAP-3a and RK-11c with a stop codon at amino acid 524; and CK-1c and KT-1c with in-frame deletions causing the loss of amino acids 280–315 (Fig. 1 and Table 2).

Sequencing of gDNA fragments from five resistant larvae from India revealed that five out of the six different cDNA mutations examined involved mis-splicing of pre-mRNA (Table 3). Four entail introduction of 5′ and/or 3′ alternative splice sites and the fifth is skipping of exon 6 described above (Table 3, Supplementary Figs S8–11). In each of these cDNA mutations, except for skipping of exon 6, the corresponding gDNA had splice-site mutations that either disrupted the native splice site or introduced alternative splice sites (Table 3). For example, in one larva (GAP-3), the native 5′ GT dinucleotide splice site of intron 8 was changed to AT, causing the splicing machinery to use a cryptic GT splice site within exon 8 (Supplementary Fig. S9 and Table 3). In two larvae (CK-1 and RK-1), a large DNA insertion within exon 4 introduced several alternative splice sites (Table 3, Supplementary Figs S8 and S11). Only one of the six different cDNA mutations examined did not involve mis-splicing of pre-mRNA (Table 3). This mutation has the same 5-bp deletion in exon 17 in both gDNA and cDNA (Table 3 and Supplementary Fig. S12).

## Discussion

The results reported here enable the first direct comparison of the genetic basis of laboratory- and field-selected resistance to Cry2Ab, which is deployed widely in Bt cotton and Bt corn. We discovered that mutations disrupting the ABC transporter gene *PgABCA2* are associated with resistance to Cry2Ab in both laboratory- and field-selected pink bollworm, a global pest of cotton. Genetic linkage analysis revealed that >10,000-fold resistance to Cry2Ab in a laboratory-selected strain from Arizona (Bt4-R2) is tightly linked with a disruptive indel mutation (*r*_*A1*_) in *PgABCA2*. In field-selected Cry2Ab-resistant pink bollworm from India, we did not find this mutation, but all of the 24 transcript variants from eight larvae collected from five field sites showed severe disruption of *PgABCA2*, including 19 distinct variants with one to three mutations each. Moreover, the most common cDNA mutation in field-selected populations from India, which entails mis-splicing that omits exon 6 and introduces a stop codon at amino acid 373, also occurred in Bt4-R2. This was the only mutation shared between Arizona and India. The other mutations were mostly from splice-site mutations that lead to mis-splicing of *PgABCA2* and were more diverse in India. The differences in *PgABCA2* resistance mutations between India and Arizona could reflect the difference in geographic origin, laboratory versus field selection, or both.

Mis-splicing of *PgABCA2* pre-mRNA was prevalent in Cry2Ab-resistant larvae from both field- and laboratory-selected pink bollworm. Each of the eight different mutations in resistant larvae from Arizona involves mis-splicing of *PgABCA2* (Table 1). For India, we found 17 different cDNA mutations in eight resistant larvae (Table 2). Evaluation of the corresponding gDNA sequences for 6 of these 17 cDNA mutations revealed that all but one are caused by mis-splicing (Table 3). Previous work showed that mis-splicing of cadherin pre-mRNA is associated with pink bollworm resistance to Bt toxin Cry1Ac in laboratory- and field-selected pink bollworm^30,31^. Also, in laboratory-selected *H. armigera*, mis-splicing of cadherin and ABCC2 is associated with resistance to Cry1Ac^32–34^. Together with the previous data, our new results suggest that mis-splicing may be a common underlying cause of both field- and laboratory-selected resistance to Bt toxins.

Similar to the results here revealing resistance to Cry2Ab is associated with mutations in *PgABCA2* in both laboratory- and field-selected pink bollworm, previous work shows laboratory-selected resistance to Cry2Ab is associated with the homologous genes in *H. armigera* and *H. punctigera* from Australia^28^. In Australia, where field populations remain susceptible to Cry2Ab^35^, the three resistance alleles detected in *H. armigera* result in premature truncation of ABCA2, while the sole reported *H. punctigera* resistance allele has a 14-bp deletion that causes missense mutations^28^. Like the prevalent role of mis-splicing in pink bollworm resistance to Cry2Ab, our analysis of the previously reported sequences^28^ indicates mis-splicing is also implicated in two of the three mutations in *H. armigera*. *Ha2Ab-R01* has an 8-bp insertion at the 5′-end of exon 16 and complete skipping of this exon while *Ha2Ab-R02* has a 5-bp deletion at the 5′-end of exon 18 and involves an alternative 5′ splice site. Using CRISPR/Cas9 to edit *HaABCA2* in *H. armigera*, each of the two introduced frameshift mutations caused >100-fold resistance to Cry2Ab^36^. This gene editing also eliminated binding of Cry2Ab to brush border membrane vesicles of *H. armigera*, demonstrating that ABCA2 is required for such binding^36^.

In addition to the association between resistance to Cry2Ab and mutations in *ABCA2* in three species of Lepidoptera noted above, resistance to Bt toxins in two other families is linked with mutations in two other ABC transporter proteins. Resistance to Cry1 toxins is associated with mutations disrupting *ABCC2* in eight lepidopteran pests^37–41^ and resistance to Cry3Aa is genetically linked with a 4-bp deletion in *ABCB1* in the coleopteran *Chrysomela tremula*^42^. The association between resistance to Cry toxins and mutations affecting ABC transporters in at least 11 species of insects indicates a key role for these proteins in the Cry toxin mode of action, which may include directly binding toxin or promoting toxin oligomerization and pore formation^37,41,43–47^.

Results from previous work related to this study show that in both laboratory-selected strains from Arizona and field-selected pink bollworm populations from India, resistance to Cry1Ac is associated with mutations disrupting a gene encoding a cadherin protein (PgCad1) that binds this toxin in the midgut of susceptible larvae^30,48–50^. Similar to the results with Cry2Ab reported here, the mutations affecting *PgCad1* were more diverse in the field-selected populations from India than in several lab-selected strains from Arizona. Whereas only four mutant cadherin alleles were found in pink bollworm from five laboratory-selected strains from Arizona, eight cadherin resistance alleles and 19 transcript isoforms were identified from just eight individuals collected from two field-selected populations in India^30^. Thus, both analyses support the conclusion that laboratory selection may be useful for identifying the genes conferring resistance to Bt toxins, but the resistance alleles are likely to be more diverse in field populations. Unlike the results here with Cry2Ab, none of the Cry1Ac resistance mutations in pink bollworm were shared between Arizona and India.

The diverse mutations in *PgABCA2* and *PgCad1* associated with resistance to Cry2Ab and Cry1Ac, respectively, in field-selected populations of pink bollworm imply that it would not be efficient to screen for specific resistance alleles, as has been done previously^51,52^. Moreover, monitoring by screening DNA would miss the critical mis-splicing variants associated with resistance to Bt toxins in this study and in previous work.

Conversely, monitoring methods such as the F_1_ screen, which can readily identify field-collected individuals with diverse and previously unknown resistance mutations, could be especially useful. In the F_1_ screen, each field-collected adult is allowed to mate in a single pair with an adult from a strain that is homozygous for a recessive resistance mutation^16,53^. This approach can detect any recessive resistance alleles in the field-collected adults that occur at the same locus as the recessive mutations in the laboratory-selected strain, as well as non-recessive mutations at any locus^15,16^. In addition to the F_1_ screen, the use of next generation sequencing is promising for monitoring variants associated with resistance in the field. This approach allowed us to multiplex cDNA samples from 22 individuals and obtain sequencing information from essentially single molecules of full-length *PgABCA2* cDNA without post-sequencing assembly. Furthermore, long read sequencing identified five *PgABCA2* transcript variants (Bt4-R2e-i) not previously found by cloning and Sanger sequencing. Even though we identified several *PgABCA2* mutations in gDNA from resistant larvae from India using traditional PCR, cloning, and Sanger sequencing, this method is laborious and not practical for monitoring the diverse mutations in the field. Given that *PgABCA2*-mediated resistance to Cry2Ab occurred in pink bollworm populations from Arizona and India, long read sequencing focusing on this gene could provide a valuable alternative to the F_1_ screen for monitoring resistance to Cry2Ab in this cosmopolitan pest.

Whereas refuges of non-Bt host plants have helped to delay pest resistance to Bt crops generally, and particularly for the pink bollworm in the United States and China^12,54^, the scarcity of such refuges probably contributed to the rapid evolution of pink bollworm resistance to both Cry1Ac and Cry2Ab in India^21,55,56^. Currently, in some regions of India, no Bt toxins in commercialized transgenic cotton are effective against pink bollworm^20,21^. However, the genetically modified toxins Cry1AbMod and Cry1AcMod are effective against laboratory-selected strains of pink bollworm from Arizona resistant to Cry1Ac and Cry2Ab^57,58^. Because the loci harboring mutations conferring resistance to these toxins are the same in laboratory- and field-selected pink bollworm, we hypothesize that these modified toxins would also kill field-selected resistant larvae from India. Nonetheless, Bt cotton producing these modified toxins is not available and no transgenic cotton effective against resistant pink bollworm in India is likely to be available in the next several years. This difficult situation underscores the value of proactively implementing resistance management to sustain the efficacy of Bt crops.

## Materials and Methods

### Insect Strains and Rearing

We used three strains of pink bollworm from Arizona: APHIS-S, AZP-R and Bt4-R2. APHIS-S is a susceptible strain that has been reared in the laboratory for more than 30 years without exposure to Bt toxins or other insecticides^59,60^. AZP-R, derived from 10 populations collected from Arizona cotton fields in 1997, has 1,500–3,100-fold resistance to Cry1Ac conferred primarily by the *r2* cadherin allele, but only two-fold resistance to Cry2Ab^61,62^. Relative to APHIS-S, Bt4-R2 had 28-fold resistance to Cry1Ac conferred by the mutant cadherin allele *r4*, and >10,000-fold resistance to Cry2Ab^27,63^. Bt4-R2 was derived from the Bt4R strain and selected in the laboratory for resistance to Cry2Ab^27^. In Bt4-R2, inheritance of resistance to Cry1Ac (10 micrograms Cry1Ac per ml diet) and Cry2Ab (0.1, 0.3, 1 and 10 microgram Cry2Ab per ml diet) was recessive based on results of 21-day diet bioassays^27,50^. All larvae were reared on wheat germ diet^64^ at 26 °C with 14 h light:10 h dark.

### Genetic Linkage Analysis and Diet Bioassays

We tested the genetic linkage between the *PgABCA2 r*_*A1*_ allele and resistance to Cry2Ab by exploiting the biphasic nature of the genetic linkage in Lepidoptera^65^. We first set up 40 F_0_ single-pair crosses between APHIS-S (*s*_*A1*_*s*_*A1*_) and Bt4-R2 (*r*_*A1*_*r*_*A1*_) for both ♀ Bt4-R2 x ♂ APHIS-S and ♀ APHIS-S x ♂ Bt4-R2 reciprocal crosses. From each cross, F_1_ eggs were collected and neonates were reared individually on untreated artificial diet until pupation. To generate backcross families, surviving F_1_ pupae (n = 22 from ♀ Bt4-R2 x ♂ APHIS-S and n = 20 from ♀ APHIS-S x ♂ Bt4-R2) were sexed and paired with a Bt4-R2 pupa of the opposite sex. A total of 5 informative F_1_ backcross families were chosen for each reciprocal cross (A, B, C, D and E from ♀ Bt4-R2 x ♂ APHIS-S and F, G, H, I and J from ♀ APHIS-S x ♂ Bt4-R2). We used diet incorporation bioassays to evaluate the susceptibility of neonates from backcross families on either untreated diet or on 1 microgram Cry2Ab per mL diet^27,62^. After 12 d at 26 °C, we scored all insects for survival and weighed the live insects. Cry2Ab protoxin from Jie Zhang [as described in Fabrick *et al*.^27^ was used for all bioassays.

### RNA Extraction and cDNA Synthesis

We extracted total RNA from pink bollworm using TRI Reagent (Invitrogen-Life Technologies, Carlsbad, CA) and cDNA was prepared using 1 μg of total RNA and random hexamer primers with the ThermoScript RT-PCR system (Invitrogen-Life Technologies) according to the manufacturer’s instruction. RNA used for PCR and cloning was from dissected alimentary tracts pooled from either 3 or 10 fourth instar larvae.

### PCR Amplification of *PgABCA2* cDNA

Prior to the availability of a pink bollworm midgut transcriptome^66^, we used degenerate PCR to amplify the *PgABCA2* cDNA sequence. Degenerate oligonucleotide primers (Supplementary Table S1) were designed using the consensus degenerate hybrid oligonucleotide primer (CODEHOP) strategy^67^ from eight different insect ABCA family members, including *Aedes aegypti* (XP_001662816), *Anopheles sinensis* (KFB51284), *Bombyx mori* (ALE60402), *Culex quinquefasciatus* (XP_001851807), *Drosophila ananassae* (XP_001967055), *Danaus plexippus* (EHJ70360), *Helicoverpa armigera* (ALF46272), and *Tribolium castaneum* (XP_008199153). Sequences were aligned using Clustal Omega (http://www.ebi.ac.uk/Tools/msa/clustalo/), and conserved blocks were prepared using the Block Maker software (http://blocks.fhcrc.org/blocks/make_blocks.html). Blocks were then used by CODEHOP program (https://virology.uvic.ca/virology-ca-tools/j-codehop/) to design degenerate primers with the default parameters. All primers were purchased from Integrated DNA Technology (IDT, Coralville, IA).

We used the primer combinations 1pgABCA2-5 + 8pgABCA2-3 and 4pgABCA2-5 + 27pgABCA2-3 (Supplementary Table S1) to amplify two internal *PgABCA2* cDNA fragments from APHIS-S. PCR was performed with 2.5 U Takara ExTaq Premix (Takara Bio USA Inc., Mountain View, CA), 2 μM of each sense and antisense primer, and 0.3 µg cDNA using a Biometra TProfessional gradient Thermocycler (Biometra, Germany) at: 95 °C for 3 min (1 cycle); 40 cycles of 95 °C for 45 s, 50 °C for 1 min and 72 °C for 2 min; then 72 °C for 5 min. Nested PCR amplification was carried using one microliter of primary PCR product using the primer combinations 2pgABCA2-5 + 6pgABCA2-3 and 15pgABCA2-5 + 23pgABCA2-3, respectively (Supplementary Table S1). PCR products cloned into pCR^®^2.1-TOPO^®^ (Invitrogen-Life Technologies) were sequenced by the Arizona State University DNA Lab (Tempe, AZ) and confirmed as *PgABCA2* using BLAST (https://blast.ncbi.nlm.nih.gov/Blast.cgi).

### Rapid Amplification of cDNA Ends (RACE)

The partial cDNA sequences obtained from degenerate PCR allowed for design of *PgABCA2*-specific sense and antisense primers (Supplementary Table S1) and were used for 5′- and 3′-RACE according to Invitrogen’s GeneRacer™ kit protocol. For 5′-RACE, cDNA was reverse transcribed using supplied random hexamer primer from 1 µg of total RNA from APHIS-S and used as templates for PCR. Semi-nested PCR amplification was used, including a first round with the GeneRacer™ 5′ Primer and 32pgABCA2-3 and the second using GeneRacer™ 5′ Nested Primer and 32pgABCA2-3. For 3′-RACE, APHIS-S total RNA was reverse transcribed using GeneRacer™ Oligo-dT Primer and nested PCR was run using 37pgABCA2-5 and the GeneRacer™ 3′ Primer followed by fully-nested amplification using 38pgABCA2-5 and the GeneRacer™ 3′ Nested Primer. PCR products were cloned into pCR^®^2.1-TOPO® and sequenced as indicated above.

### Sanger Sequencing of the Full-Length *PgABCA2* Coding Region

Oligonucleotide primers, 55pgABCA2-5 and 60pgABCA2-3 (Supplementary Table S1), corresponding to the 5′- and 3′-ends of *PgABCA2* were used to amplify the full-length coding sequence from APHIS-S cDNA described earlier. PCR was performed using 1.25 U SuperTaq^TM^ DNA polymerase (Applied Biosystems, Foster City, CA) and 0.4 μM primers at: 94 °C for 2 min (1 cycle); 40 cycles of 94 °C for 30 s, 60 °C for 30 s and 68 °C for 6 min; then 72 °C for 5 min. PCR products were A-tailed with 1 U of Takara ExTaq and separated by agarose gel electrophoresis. A band of ~5 kb was gel-purified and multiple attempts were made to clone into several cloning vectors optimized for cloning long PCR amplicons and to minimize “leaky” expression of genes. Unfortunately, no *PgABCA2*-positive *E. coli* clones were obtained, suggesting that the insert may be cytotoxic to bacteria.

Because of difficulties with cloning the full-length *PgABCA2* coding region, we verified the 55pgABCA2-5 + 60pgABCA2-3 PCR products by direct sequencing. PCR products were treated with ExoSAP-IT™ PCR Product Cleanup Reagent (Affymetrix, Inc. Santa Clara, CA) according to manufacturer’s protocol and sequenced directly with oligonucleotide primers, 63pgABCA2-3 through 78pgABCA2-5 (Supplementary Table S1). Similarly, we amplified the full-length coding sequence from cDNA prepared from Bt4-R2 and PCR products were directly sequenced and compared with the APHIS-S *PgABCA2* sequence. The full-length coding sequence of *PgABCA2* from APHIS-S, AZP-R, and Bt4-R2 are deposited in the GenBank public database (MG637361).

To further validate differences within the *PgABCA2* cDNA sequences from APHIS-S and Bt4-R2, three overlapping, partial fragments corresponding to nucleotides 1–1,744, 1,656–3,403, and 3,326–5,187 were PCR amplified and cloned into pCR^®^2.1-TOPO^®^. Several clones corresponding to each fragment were Sanger sequenced. The *PgABCA2* cDNA sequences that were unique after sequencing these clones were identified as “variants”. We used Mutalyzer 2.0.26 (https://mutalyzer.nl/description-extractor)^68^ and Description Extractor^69^ to detect all sequence changes (≥2 bp) found within the Bt4-R2 cDNA variants and all mutations were annotated based on nomenclature and recommendations provided by Human Genome Variation Society (http://www.HGVS.org/varnomen)^70^. Changes resulting from single nucleotide polymorphisms were excluded from our analysis.

### Extraction of gDNA, PCR and Cloning

gDNA was extracted from pools of ten APHIS-S and Bt4-R2 using the PUREGENE DNA Isolation Kit (Qiagen, Valencia, CA) according to the manufacturer’s instructions. We extracted gDNA from the remaining tissues (e.g. larvae minus gut tracts) from the same 10 individuals that were used for RNA extractions. gDNA was similarly extracted from India and Arizona individuals for PCR genotyping and Sanger sequencing.

Because exon-intron junctions are unknown for the *PgABCA2* gene, we used Splign, (https://www.ncbi.nlm.nih.gov/sutils/splign/splign.cgi)^71^ and the *H. armigera* ABCA2 coding sequence (KP219763) with its corresponding genomic locus (NW_018395393.1) to predict intron positions in *PgABCA2*. The primers 91pgABCA2-5 and 92pgABCA2-3 (Supplementary Table S1) located in the exons flanking the *r*_*A1*_ mutation were used to amplify genomic fragments from APHIS-S and Bt4-R2 and the gDNA products were cloned into pCR®2.1-TOPO® and sequenced as above. To check genomic DNA corresponding to exon 6 region (1,090-1,234 bp) in Bt4-R2, two different sets of primers were used (143pgABCA2-5 + 106pgABCA2-3 and 95pgABCA2-5 + 90pgABCA2-3).

### Allele-Specific PCR

Based on the location of the *r*_*A1*_ mutation in the gDNA, we used allele-specific PCR and primers 83pgABCA2-5 and 84pgABCA2-3 (hereafter referred to as r_A1_-F and r_A1_-R) (Supplementary Table S1) that flank the *r*_*A1*_ mutation to genotype backcross survivors obtained from biphasic linkage crosses. Reactions contained 0.1 μg gDNA, 2X SaphireAmp^®^ polymerase premix (Takara Bio USA Inc., Mountain View, CA), and 0.2 μM of both sense and antisense primers. PCR was run using the following conditions: 94 °C for 1 min (1 cycle); 25 cycles of 98 °C for 5 s, 60 °C for 5 s and 72 °C for 5 s. PCR products and 1 kb Plus DNA Ladder (ThermoFisher Scientific) were separated using 3% agarose gel electrophoresis to differentiate size of mutant (*r*_*A1*_) and wild-type (*s*_*A1*_) DNA bands. To validate allele-specific PCR, amplified products were cloned and sequenced from Bt4-R2 (n = 4) and APHIS-S (n = 4).

### Targeted *PgABCA2* Sequencing and Bioinformatics Analysis

We used Single Molecule, Real-Time (SMRT^®^) sequencing on a PacBio^®^ RSII (Pacific Biosciences, Menlo Park, CA) to sequence *PgABCA2* cDNA from 22 pink bollworm larvae. Fifteen larvae were survivors obtained from backcross families on 1 microgram Cry2Ab per mL diet, including 7 genotyped by allele-specific PCR as *r*_*A1*_*r*_*A1*_ and 8 genotyped as *r*_*A1*_*s*_*A1*_. APHIS-S larvae were taken directly from our rearing colony on November 17, 2016.

Total RNA was extracted and cDNA was prepared from individual 4^th^ instar larvae (excluding head) as previously described, except we used a gene-specific primer (150pgABCA2-3; Supplementary Table S1) to enrich the cDNA for *PgABCA2*. Because of limited available tissue and low abundance of the *PgABCA2* transcript, we used nested PCR to generate amplicon templates for library preparation. Initial PCR amplification was performed with primers 158pgABCA2-5 and 150pgABCA2-3 (Supplementary Table S1) using SuperTaq^TM^ DNA polymerase (Applied Biosystems). Nested amplification was carried out with barcode-tailed PCR primers, 163pgABCA2-5 and 166pgABCA2-3 (Supplementary Table S1). The symmetric barcode-tailed PCR primers were designed based on the PacBio^®^ multiplex PCR primer guidelines (http://www.2einteractive.com/pacbio/Shared-Protocol-PacBio-Barcodes-for-SMRT-Sequencing.pdf).

Prior to SMRTbell^TM^ library preparation, ~5 kb PCR products were gel purified with QIAquick^®^ Gel extraction kit (Qiagen, Germantown, MD) and quantified using Qubit^TM^ dsDNA High Sensitivity Assay Kit with the Qubit^TM^ 2.0 Fluorometer (ThermoFisher Scientific). Barcoded samples (n = 22) were pooled in equimolar concentrations and SMRTbell^TM^ libraries were prepared with 0.5 µg of the pooled amplicons following the standard procedures for blunt ligation of hairpin adapters using SMRTbell^TM^ Template Prep Kit 1.0 (Pacific Biosciences). DNA sequencing was performed on the PacBio^®^ RSII sequencer using P6-P4 enzyme chemistry at the University of Arizona, Arizona Genomics Institute (Tucson, Arizona). Data were captured using 3-hour movies. Sequence data are deposited in the NCBI TSA under the accession SRP126193, associated with BioProject PRJNA421207.

We initially processed the raw sequences through the PacBio SMRT^®^ portal (v2.3.0), in which sequences were filtered for a minimum of two passes and a minimum predicted accuracy of 90%. We used Long Amplicon Analysis (LAA v2) protocol to identify and report abundance of differing clusters of sequencing reads within a single library. Custom LAA settings included: minimum subread length = 4,000; maximum number of subreads = 1000; barcode score = 30; ignore primer sequence = 30; trim ends = 0; only most supported = 0; cluster per gene family = y; phase alleles = y; split results = y. We also processed the raw sequences by LAA analysis using slightly modified settings to include shorter subreads (minimum subread length = 3000). The latter analysis allowed us to sample more reads and to specifically identify reads containing the exon 6 deletion. Resulting consensus sequences were manually inspected (for length and subread coverage to exclude spurious artifact sequences) and mapped to the *PgABCA2* cDNA plus strand using Geneious R10 (Biomatters Inc., Newark, NJ).

### Pink Bollworm Field Collections

We analyzed putatively resistant pink bollworm larvae collected from December 11, 2015 to January 3, 2016 at seven sites in four states of India (Fig. 4). Fourth instars were collected by opening mature cotton bolls collected arbitrarily from cotton fields reported to be planted with dual-toxin Bt cotton producing Cry1Ac and Cry2Ab. Fourth instar larvae were saved in RNAlater^®^ (Ambion-Life Technologies, Carlsbad, CA). We used EnviroLogix QuantiPlate^TM^ ELISA kits (Portland, ME) for Cry1Ab/Cry1Ac and Cry2A to estimate the concentration of Cry1Ac and Cry2Ab in maternal tissue from cotton bolls for which survivors were recovered. ELISAs were conducted according to manufacturer’s protocols on boll material stored frozen at −20 °C for 2–4 months prior to analysis. Toxins provided with the kit were used as positive controls, single samples were run, and concentrations were expressed as micrograms Cry toxin per gram wet tissue.

### DNA Screening of Populations from India for *PgABCA2 r*_*A1*_ Allele

We extracted gDNA from the head portion of seven pink bollworm survivors on Bollgard II^®^ that remained following RNA extraction and performed *r*_*A1*_-specific PCR as described above. PCR products were separated on 3% agarose gels and visually inspected for *PgABCA2* product size different from control samples (APHIS-S and Bt4-R2).

### Sequencing of *PgABCA2* DNA from Field-Collected Resistant and Susceptible Pink Bollworm from India

Genomic DNA, total RNA and cDNA were prepared from pink bollworm recovered from Bollgard II^®^ cotton bolls from India as described above. We extracted total RNA from 8 individuals (AM-8, CK-1, GAP-3, KT-1, KT-8, KT-10, RK-1 and RK-11), and cDNA was synthesized using the *PgABCA2*-specific primer, 60pgABCA2-3. cDNA from three pink bollworm samples (AMH-1, AMH-2 and AMH-3) collected 3 November 2010 on non-Bt cotton bolls at the Panjabrao Deshmukh Agricultural University Cotton Research Station in Akola, Maharashtra served as susceptible control samples^30^. Full-length *PgABCA2* cDNA was first amplified using 55pgABCA2-5 and 60pgABCA2-3 primers and SuperTaq^TM^ DNA polymerase (Applied Biosystems) followed by nested amplification with 104pgABCA2-5 and 105pgABCA2-3. PCR products were separated by 1% agarose gel electrophoresis to confirm presence of a single amplicon and remainder of samples were used for Sanger sequencing of *PgABCA2* PCR products using sequencing primers described above.

*PgABCA2* PCR sequence reads were mapped to the reference *PgABCA2* cDNA sequence from AMH using Geneious R10 and variation was observed in traces showing multiple overlapping sequencing. We amplified and cloned cDNAs corresponding to these variable regions into pCR^®^2.1-TOPO^®^ or pCR-XL-TOPO^®^ and plasmid DNA was sequenced. Specifically, for CK-1 and GAP-3, 61pgABCA2-5 and 125pgABCA2-3 amplified a fragment corresponding to putative exons 2–18. For samples KT-1, KT-8, KT-10, RK-1 and RK-11 samples, overlapping fragments were amplified using primers 104pgABCA2-5 and 169pgABCA2-3 and 70pgABCA2-5 and 169pgaBCA-3 spanning exons 1–16 and 13–31, respectively. The *PgABCA2* cDNA variants from India that differed by ≥2 bp and unique from the AMH consensus sequence were further analyzed using Mutalyzer and were annotated based on nomenclature and recommendations provided by the Human Genome Variation Society^70^. We checked the gDNA regions that overlapped with some mutations from several of the putative resistant samples from India (AM-8, CK-1, GAP-3, KT-1 and RK-1). To amplify the gDNA, we used primers 124pgABCA2-5 and 125pgABCA2-3 for AM-8, 191pgABCA2-5 and 192pgABCA2-3 for CK-1, 174pgABCA2-5 and 185pgABCA2-3 for GAP-3, 49pgABCA2-5 and 89pgABCA2-3, 104pgABCA2-5 and 89pgABCA2-3, 64pgABCA2-5 and 120pgABCA2-3, and 121pgABCA2-5 and 123pgABCA2-3 for RK-1 (Supplementary Table S1). To amplify a common region overlapping the mis-splicing observed in exon 6, primers 96pgABCA2-5 and 90pgABCA2-3 were used to amplify gDNA from CK-1, KT-1 and RK-1 (Supplementary Table S1). The partial gDNA sequences were cloned into pCR^®^2.1-TOPO® or pCR-XL-TOPO^®^ and sequenced.

## Electronic supplementary material

Supplementary Information
