# Supplementary material for: ABC transporter mis-splicing associated with resistance to Bt toxin Cry2Ab in laboratory- and field-selected pink bollworm
Source: Sci Rep. 2018 Sep 10;8:13531. doi: 10.1038/s41598-018-31840-5 (PMC6131251; doi:10.1038/s41598-018-31840-5)

SUPPLEMENTARY INFORMATION

ABC transporter mis-splicing associated with resistance to Bt toxin Cry2Ab in laboratory- and field-selected pink bollworm

Lolita G. Mathew[1], Jeyakumar Ponnuraj[2], Bheemanna Mallappa[3], Lingutla R. Chowdary[4], Jianwei Zhang[5], Wee Tek Tay[6], Thomas K. Walsh[6], Karl H. J. Gordon[6], David G. Heckel[7], Sharon Downes[8], Yves Carrière[9], Xianchun Li[9], Bruce E. Tabashnik[9], and Jeffrey A. Fabrick[1,a]

[1] U.S. Department of Agriculture (USDA), Agricultural Research Service (ARS), U.S. Arid Land Agricultural Research Center, Maricopa, AZ 85138, USA
[2] Indian Council of Agricultural Research (ICAR), Indian Institute of Rice Research (IIRR), Rajendra Nagar, Hyderabad, 500 030, India
[3] Pesticide Residue and Food Quality Analysis Laboratory, University of Agricultural Sciences, Raichur, Karnataka, 584 104, India
[4] Agriculture Research Station, Acharya N.G. Ranga Agricultural University, Darsi, Andhra Pradesh, 523 247, India
[5] Arizona Genomics Institute, University of Arizona, Tucson, AZ 85721, USA
[6] Commonwealth Scientific and Industrial Research Organization (CSIRO), Black Mountain Laboratories, Acton, ACT 2601, Australia
[7] Department of Entomology, Max Planck Institute for Chemical Ecology, 07745 Jena, Germany
[8] Commonwealth Scientific and Industrial Research Organization (CSIRO), Myall Vale Laboratories, Narrabri, NSW 2390, Australia
[9] Department of Entomology, University of Arizona, Tucson, AZ 85721, USA

[a] Corresponding author:
Jeffrey A. Fabrick
USDA ARS, U.S. Arid Land Agricultural Research Center
21881 N. Cardon Lane
Maricopa, AZ 85138, USA
Phone: 520-316-6335
Email: jeff.fabrick@ars.usda.gov

SUPPLEMENTARY INFORMATION includes:

Supplementary Tables S1-S4

Supplementary Figure Legends

Supplementary Figures S1-S13

Supplementary Table S1. Nucleotide primers used to amplify, genotype, and/or DNA sequence *PgABCA2*.

Primer	Sequence	Direction	Application
1pgABCA2-5	5' - CATCACCGTAGAAAAAGAAAAGcarytnaarga - 3'	Sense	degenerate primer
8pgABCA2-3	5' - CTCGTCGAGCGGATggrtccatncc - 3'	Antisense	degenerate primer
4pgABCA2-5	5' - TTGTACTGTGCGATGAGCCTACTdsnggnatgga - 3'	Sense	degenerate primer
27pgABCA2-3	5' - AGGGTGAAACCCTTGGAGAayttrttyttna - 3'	Antisense	degenerate primer
2pgABCA2-5	5' - GAAAAAGAAAAGCAACTGAAGGAArynatgaarat - 3'	Sense	degenerate primer
6pgABCA2-3	5' - CGATGCCAACGCATAGTTTTckyttywkncc - 3'	Antisense	degenerate primer
15pgABCA2-5	5' - GGCGGGAAAGACAACCacnttyaarat - 3'	Sense	degenerate primer
23pgABCA2-3	5' - GGAACCTAGACACTTGAACTCACcrttnaccatda - 3'	Antisense	degenerate primer
32pgABCA2-3	5' - TCGCTTTAGGCCGCCTGATAACCCCGC - 3'	Antisense	5' RACE
37pgABCA2-5	5' - TCCAGCGGAGATGCCTTCGTGAGTGG - 3'	Sense	3' RACE
38pgABCA2-5	5' - AGCGGAGATGCCTTCGTGAGTGGCC - 3'	Sense	3' RACE
63pgABCA2-3	5' - CGCCTGTCATGAAGAAGGA - 3'	Antisense	PCR product sequencing
64pgABCA2-5	5' - ATTGCGTGAAATAACGGAGC - 3'	Sense	PCR product sequencing, gDNA PCR amplification of RK-1
65pgABCA2-3	5' - CCGCTGTACTGGCTTTTGA - 3'	Antisense	PCR product sequencing
66pgABCA2-5	5' - ACCTTGGACCGTCCTCTTCT - 3'	Sense	PCR product sequencing
67pgABCA2-3	5' - GTCCACGTTACCTGTGAGCA - 3'	Antisense	PCR product sequencing
68pgABCA2-5	5' - TTTACGACGACCAAATCACG - 3'	Sense	PCR product sequencing
69pgABCA2-3	5' - TCACAACAGTTCCAGGGATG - 3'	Antisense	PCR product sequencing
70pgABCA2-5	5' - GGAGTCGGCTACACGCTAGT - 3'	Sense	PCR product sequencing
71pgABCA2-3	5' - TCCATTGTGGGATTATTGGC - 3'	Antisense	PCR product sequencing
72pgABCA2-5	5' - GTTTCAAAGGGTTGTCCCCT - 3'	Sense	PCR product sequencing
73pgABCA2-3	5' - CCTATCGGTAGACTGGCGG - 3'	Antisense	PCR product sequencing
74pgABCA2-5	5' - AACGTCATTGGGCTCTCTTC - 3'	Sense	PCR product sequencing
75pgABCA2-3	5' - CCATAATACTTGGTGAGGCCA - 3'	Antisense	PCR product sequencing
76pgABCA2-5	5' - CACAATGTTGCATCCCAGAC - 3'	Sense	PCR product sequencing

77pgABCA2-5	5' - GCAATTCGAAGCGACATTC - 3'	Sense	PCR product sequencing
78pgABCA2-5	5' - CGATGCTGTGAAGCATTACG - 3'	Sense	PCR product sequencing
55pgABCA2-5	5' - ATGCGGGCGCGTGGAGAGC - 3'	Sense	full length coding sequence 5'-primer; 1st fragment
60pgABCA2-3	5' - TTATGTCTGTTGTGCTTCATGTTGATACTTTGTGAA - 3'	Antisense	full length coding sequence 3'-primer; 3rd fragment
56pgABCA2-3	5' - TGTTTACCGCAACGTTGCTCCCGTAAAC - 3'	Antisense	1st fragment
57pgABCA2-5	5' - AACAAAAGAAAGCGACCCTAC - 3'	Sense	2nd fragment
58pgABCA2-3	5' - TGATGAACACAGCACTGACAA - 3'	Antisense	2nd fragment
59pgABCA2-5	5' - CAATGGTAGCGTTCCTCTCGATGCAA - 3'	Sense	3rd fragment
83pgABCA2-5 (r_{A1}-F)	5' - CGCCAAGCTGCTGCAGAAG - 3'	Sense	Allele specific PCR
84pgABCA2-3 (r_{A1}-R)	5' - TGAGTCGTCCAACTAATCCTGACTGATAACAT - 3'	Antisense	Allele specific PCR
91pgABCA2-5	5' - CAGCCAACTTGACTGTTTACAACCA - 3'	Sense	gDNA PCR amplification of Bt4-R2
92pgABCA2-3	5' - GCAGGGCCTTTGAAGAAATAGGA - 3'	Antisense	gDNA PCR amplification of Bt4-R2
150pgABCA2-3	5' - CGTGTTAAATAATTCCACCGCCATTG - 3'	Antisense	GSP 3'-primer for RT reaction and PCR
158pgABCA2-5	5' - CCCGAGTTATAGTGTGTACCTTC - 3'	Sense	PacBio 1st PCR amplification
163pgABCA2-5_0002_Forward	5' - CTATACATGACTCTGCGAATTTGGATTTGAGGCG - 3'	Sense	PacBio nested forward primer with barcode
163pgABCA2-5_0003_Forward	5' - TACTAGAGTAGCACTCGAATTTGGATTTGAGGCG - 3'	Sense	PacBio nested forward primer with barcode
163pgABCA2-5_0004_Forward	5' - TGTGTATCAGTACATGGAATTTGGATTTGAGGCG - 3'	Sense	PacBio nested forward primer with barcode
163pgABCA2-5_0006_Forward	5' - GATCTCTACTATATGCGAATTTGGATTTGAGGCG - 3'	Sense	PacBio nested forward primer with barcode
163pgABCA2-5_0007_Forward	5' - ACAGTCTATACTGCTGGAATTTGGATTTGAGGCG - 3'	Sense	PacBio nested forward primer with barcode
163pgABCA2-5_0008_Forward	5' - ATGATGTGCTACATCTGAATTTGGATTTGAGGCG - 3'	Sense	PacBio nested forward primer with barcode
163pgABCA2-5_0014_Forward	5' - CGTCTATATACGTATAGAATTTGGATTTGAGGCG - 3'	Sense	PacBio nested forward primer with barcode
163pgABCA2-5_0015_Forward	5' - ATAGAGACTCAGAGCTGAATTTGGATTTGAGGCG - 3'	Sense	PacBio nested forward primer with barcode
163pgABCA2-5_0016_Forward	5' - TAGATGCGAGAGTAGAGAATTTGGATTTGAGGCG - 3'	Sense	PacBio nested forward primer with barcode
163pgABCA2-5_0018_Forward	5' - CATCACTACGCTAGATGAATTTGGATTTGAGGCG - 3'	Sense	PacBio nested forward primer with barcode
163pgABCA2-5_0020_Forward	5' - TATGTGATCGTCTCTCGAATTTGGATTTGAGGCG - 3'	Sense	PacBio nested forward primer with barcode
163pgABCA2-5_0023_Forward	5' - ATATCAGTCATGCATAGAATTTGGATTTGAGGCG - 3'	Sense	PacBio nested forward primer with barcode
163pgABCA2-5_0030_Forward	5' - AGAGATGTGTGATGACGAATTTGGATTTGAGGCG - 3'	Sense	PacBio nested forward primer with barcode

163pgABCA2-5_0031_Forward	5' - TACGACTACATATCAGGAATTTGGATTTGAGGCG - 3'	Sense	PacBio nested forward primer with barcode
163pgABCA2-5_0032_Forward	5' - TATCTCTGTAGAGTCTGAATTTGGATTTGAGGCG - 3'	Sense	PacBio nested forward primer with barcode
163pgABCA2-5_0035_Forward	5' - TCTATGTCTCAGTAGTGAATTTGGATTTGAGGCG - 3'	Sense	PacBio nested forward primer with barcode
163pgABCA2-5_0039_Forward	5' - CTGTGTGTGATAGAGTGAATTTGGATTTGAGGCG - 3'	Sense	PacBio nested forward primer with barcode
163pgABCA2-5_0041_Forward	5' - GTACATATGCGTCTGTGAATTTGGATTTGAGGCG - 3'	Sense	PacBio nested forward primer with barcode
163pgABCA2-5_0042_Forward	5' - GAGACTAGAGATAGTGGAATTTGGATTTGAGGCG - 3'	Sense	PacBio nested forward primer with barcode
163pgABCA2-5_0044_Forward	5' - TGTCACTCATCTGAGTGAATTTGGATTTGAGGCG - 3'	Sense	PacBio nested forward primer with barcode
163pgABCA2-5_0049_Forward	5' - TCACATATGTATACATGAATTTGGATTTGAGGCG - 3'	Sense	PacBio nested forward primer with barcode
163pgABCA2-5_0054_Forward	5' - GTGTGAGATATATATCGAATTTGGATTTGAGGCG - 3'	Sense	PacBio nested forward primer with barcode
163pgABCA2-5_0058_Forward	5' - AGATATCATCAGCGAGGAATTTGGATTTGAGGCG - 3'	Sense	PacBio nested forward primer with barcode
166 pgABCA2-3_0002_Reverse	5' - GCAGAGTCATGTATAGTCGAGGGTAGTTTGTGAT - 3'	Antisense	PacBio nested reverse primer with barcode
166 pgABCA2-3_0003_Reverse	5' - GAGTGCTACTCTAGTATCGAGGGTAGTTTGTGAT - 3'	Antisense	PacBio nested reverse primer with barcode
166 pgABCA2-3_0004_Reverse	5' - CATGTACTGATACACATCGAGGGTAGTTTGTGAT - 3'	Antisense	PacBio nested reverse primer with barcode
166 pgABCA2-3_0006_Reverse	5' - GCATATAGTAGAGATCTCGAGGGTAGTTTGTGAT - 3'	Antisense	PacBio nested reverse primer with barcode
166 pgABCA2-3_0007_Reverse	5' - CAGCAGTATAGACTGTTCGAGGGTAGTTTGTGAT - 3'	Antisense	PacBio nested reverse primer with barcode
166 pgABCA2-3_0008_Reverse	5' - AGATGTAGCACATCATTCGAGGGTAGTTTGTGAT - 3'	Antisense	PacBio nested reverse primer with barcode
166 pgABCA2-3_0014_Reverse	5' - TATACGTATATAGACGTCGAGGGTAGTTTGTGAT - 3'	Antisense	PacBio nested reverse primer with barcode
166 pgABCA2-3_0015_Reverse	5' - AGCTCTGAGTCTCTATTCGAGGGTAGTTTGTGAT - 3'	Antisense	PacBio nested reverse primer with barcode
166 pgABCA2-3_0018_Reverse	5' - ATCTAGCGTAGTGATGTCGAGGGTAGTTTGTGAT - 3'	Antisense	PacBio nested reverse primer with barcode
166 pgABCA2-3_0020_Reverse	5' - GAGAGACGATCACATATCGAGGGTAGTTTGTGAT - 3'	Antisense	PacBio nested reverse primer with barcode
166 pgABCA2-3_0023_Reverse	5' - TATGCATGACTGATATTCGAGGGTAGTTTGTGAT - 3'	Antisense	PacBio nested reverse primer with barcode
166 pgABCA2-3_0030_Reverse	5' - GTCATCACACATCTCTTCGAGGGTAGTTTGTGAT - 3'	Antisense	PacBio nested reverse primer with barcode
166 pgABCA2-3_0031_Reverse	5' - CTGATATGTAGTCGTATCGAGGGTAGTTTGTGAT - 3'	Antisense	PacBio nested reverse primer with barcode
166 pgABCA2-3_0032_Reverse	5' - AGACTCTACAGAGATATCGAGGGTAGTTTGTGAT - 3'	Antisense	PacBio nested reverse primer with barcode
166 pgABCA2-3_0035_Reverse	5' - ACTACTGAGACATAGATCGAGGGTAGTTTGTGAT - 3'	Antisense	PacBio nested reverse primer with barcode
166 pgABCA2-3_0039_Reverse	5' - ACTCTATCACACACAGTCGAGGGTAGTTTGTGAT - 3'	Antisense	PacBio nested reverse primer with barcode
166 pgABCA2-3_0041_Reverse	5' - ACAGACGCATATGTACTCGAGGGTAGTTTGTGAT - 3'	Antisense	PacBio nested reverse primer with barcode
166 pgABCA2-3_0042_Reverse	5' - CACTATCTCTAGTCTCTCGAGGGTAGTTTGTGAT - 3'	Antisense	PacBio nested reverse primer with barcode

166 pgABCA2-3_0044_Reverse	5' - ACTCAGATGAGTGACATCGAGGGTAGTTTGTGAT - 3'	Antisense	PacBio nested reverse primer with barcode
166 pgABCA2-3_0049_Reverse	5' - ATGTATACATATGTGATCGAGGGTAGTTTGTGAT - 3'	Antisense	PacBio nested reverse primer with barcode
166 pgABCA2-3_0054_Reverse	5' - GATATATATCTCACACTCGAGGGTAGTTTGTGAT - 3'	Antisense	PacBio nested reverse primer with barcode
166 pgABCA2-3_0058_Reverse	5' - CTCGCTGATGATATCTTCGAGGGTAGTTTGTGAT - 3'	Antisense	PacBio nested reverse primer with barcode
104 pgABCA2-5	5' - AGGCGGGCTCATGGGTGAA - 3'	Sense	PCR amplification of India samples; cDNA of KT-1, KT-8, KT-10, RK-1 and RK-11 and gDNA (RK-1)
105 pgABCA2-3	5' - GAATATCTGCTCGAGGGTAGTTTGTGATA - 3'	Antisense	PCR amplification of India samples; cDNA of KT-1, KT-8, KT-10, RK-1 and RK-11
61pgABCA2-5	5' - TGAGGCATTGCCAATAGAAATACCGC - 3'	Sense	cDNA amplification of CHK-1 and GAP-3
125pgABCA2-3	5' - ATCAACAGACTGTGCCGGAGC - 3'	Antisense	cDNA amplification of CHK-1 and GAP-3
187pgABCA2-3	5' - CAACAGGATAAGCCAAGACCTTGTCA - 3'	Antisense	cDNA amplification of KT-1, KT-8, KT-10, RK-1, RK-11
70pgABCA2-5	5' - GGAGTCGGCTACACGCTAGT - 3'	Sense	cDNA amplification of KT-1, KT-8, KT-10, RK-1, RK-11
169pgABCA2-3	5' - CGCCTCCATTATGCCGAACATTCT - 3'	Antisense	cDNA amplification of KT-1, KT-8, KT-10, RK-1, RK-11
124pgABCA2-5	5' - TTCGCTAGCGAAGGCTGCCT - 3'	Sense	gDNA PCR amplification of AM-8
125pgABCA2-3	5' - ATCAACAGACTGTGCCGGAGC - 3'	Antisense	gDNA PCR amplification of AM-8
143pgABCA2-5	5' - ACTCCCAACGTGGCTGCAT - 3'	Sense	gDNA PCR amplification of Bt4-R2, RK-1
106 pgABCA2-3	5' - CTCCAATCGGAAGGTCTGTAGG - 3'	Antisense	gDNA PCR amplification of Bt4-R2, RK-1
95pgABCA2-5	5' - CAATTTATTTATTTGCTGATTGCTTCGGT - 3'	Sense	gDNA PCR amplification of Bt4-R2, RK-1
90pgABCA2-3	5' - CATAGCCAGGAGGAATGCG - 3'	Antisense	gDNA PCR amplification of Bt4-R2, CK-1, KT-1, RK-1
191pgABCA2-5	5' - GTTCGAAGTTCCCGGACCT - 3'	Sense	gDNA PCR amplification of CK-1
192pgABCA2-3	5' - GGGAGTCCCATGATCTTCATC - 3'	Antisense	gDNA PCR amplification of CK-1
96pgABCA2-5	5' - ATTTGCTGATTGCTTCGGTTTTGATAC - 3'	Sense	gDNA PCR amplification of CK-1, KT-1
174pgABCA2-5	5' - CATGGACTTGGCGAAGGAAG - 3'	Sense	gDNA PCR amplification of GAP-3
185pgABCA2-3	5' - AAGGTCTGTAGGGTCGCTTTC - 3'	Antisense	gDNA PCR amplification of GAP-3
89pgABCA2-3	5' - ATGCGGGGATATACGTCAGAAAC - 3'	Antisense	gDNA PCR amplification of RK-1
49pgABCA2-5	5' - GAAATGCGGGCGCGTGGA - 3'	Sense	gDNA PCR amplification of RK-1
120pgABCA2-3	5' - GCCATCCAATGCAGCCA - 3'	Antisense	gDNA PCR amplification of RK-1
121pgABCA2-5	5' - CACGATTCGTATTCGGTCACGTC - 3'	Sense	gDNA PCR amplification of RK-1
123pgABCA2-3	5' - GCAGAGTCCAATGTGTGAGCGT - 3'	Antisense	gDNA PCR amplification of RK-1

Supplementary Table S2. Genetic linkage between *PgABCA2* and resistance to Cry2Ab in a laboratory-selected strain of pink bollworm from Arizona (Bt4-R2).

			Bioassays of backcross (BC) progeny[a]			Genotypes of survivors from bioassays[b]		
**F_0 Family**	**F_0 female**	**F_0 male**	**BC family**	**n**	**Live**	**$r_{A1}r_{A1}$**	**$r_{A1}s_{A1}$**	**% $r_{A1}r_{A1}$**
10 backcross families with progeny produced from single-pair crosses with female F_1 (no crossing over) X male Bt4-R2								
A	Bt4-R2	APHIS-S	A-1	19	10	10	0	100
C	Bt4-R2	APHIS-S	C-2	40	15	15	0	100
E	Bt4-R2	APHIS-S	E-6	40	18	18	0	100
E	Bt4-R2	APHIS-S	E-8	40	14	14	0	100
F	Bt4-R2	APHIS-S	F-1	49	26	26	0	100
G	APHIS-S	Bt4-R2	G-2	22	8	7	1	88
H	APHIS-S	Bt4-R2	H-1	18	13	10	3	77
H	APHIS-S	Bt4-R2	H-5	19	10	8	2	80
J	APHIS-S	Bt4-R2	J-3	22	10	9	1	90
K	APHIS-S	Bt4-R2	K-1	16	10	8	2	80
**Total**				**285**	**134**	**125**	**9**	**93**
10 backcross families with progeny produced from single-pair crosses with male F_1 (crossing over) X female Bt4-R2								
B	Bt4-R2	APHIS-S	B-1	17	8	8	0	100
B	Bt4-R2	APHIS-S	B-2	35	11	11	0	100
C	Bt4-R2	APHIS-S	C-3	24	9	9	0	100
E	Bt4-R2	APHIS-S	E-4	22	11	11	0	100
E	Bt4-R2	APHIS-S	E-5	33	14	14	0	100
H	APHIS-S	Bt4-R2	H-2	20	14	10	4	71
H	APHIS-S	Bt4-R2	H-7	41	13	13	0	100
I	APHIS-S	Bt4-R2	I-1	17	10	10	0	100
I	APHIS-S	Bt4-R2	I-2	40	19	16	3	84
J	APHIS-S	Bt4-R2	J-2	22	11	11	0	100
**Total**				**271**	**120**	**113**	**7**	**94**

[a] Survivors were determined after bioassays for 12 days on diet with 1 microgram Cry2Ab per mL diet.

[b] *PgABCA2* genotypes based on allele-specific PCR (Fig 2). Backcross (BC) families originating from grandparent crosses between resistant (Bt4-R2) F_0 females and susceptible (APHIS-S) F_0 males show complete linkage between *PgABCA2* and resistance (136 $r_{A1}r_{A1}$: 0 $r_{A1}s_{A1}$). BC families originating from resistant (Bt4-R2) F_0 males and susceptible (APHIS-S) F_0 females also show strong linkage, but contain all of the recombinants (102 $r_{A1}r_{A1}$: 16 $r_{A1}s_{A1}$). The difference between the two types of F_0 crosses is not a maternal effect, because the percentage of survivors with the $r_{A1}r_{A1}$ genotype was similar for BC families in which the resistant parent was male (93%) or female (94%).

Supplementary Table S3. Barcoded PacBio® sequencing and Long Amplicon Analysis of *PgABCA2* cDNA from 22 larvae: seven APHIS-S (susceptible) and 15 survivors from the linkage experiment consisting of eight $r_{A1}r_{A1}$ and seven $r_{A1}s_{A1}$ based on genotyping by PCR.

Individual[a]	Source[b]	Total reads	Consensus reads	Changes in CDS[c]
$s_{A1}s_{A1}$-1	APHIS-S	18735	247	No
$s_{A1}s_{A1}$-2	APHIS-S	20720	500	No
$s_{A1}s_{A1}$-3	APHIS-S	46065	500	No
$s_{A1}s_{A1}$-4	APHIS-S	30507	500	No
$s_{A1}s_{A1}$-5	APHIS-S	23597	500	No
$s_{A1}s_{A1}$-6	APHIS-S	2593	-	NA[d]
$s_{A1}s_{A1}$-7	APHIS-S	3438	-	NA[d]
$r_{A1}r_{A1}$-1	G-2	29272	321	Yes (20)
			83	Yes (6; 20)[e]
			51	Yes (19-20)
$r_{A1}r_{A1}$-2	H-1	16902	235	Yes (19-20)
			108	Yes (6; 19-20)[e]
			95	Yes (20)
$r_{A1}r_{A1}$-3[f]	H-1	17857	134	Yes (20)
			91	Yes (20)
			73	Yes (20)
$r_{A1}r_{A1}$-4	H-5	23823	289	Yes (19-20)[e]
			118	Yes (20)
			92	Yes (20-22)[e]
$r_{A1}r_{A1}$-5[f]	J-3	8893	132	Yes (20-24)[e]
$r_{A1}r_{A1}$-6	K-1	3811	36	Yes (20)
$r_{A1}r_{A1}$-7	H-5	3582	-	NA[d]
$r_{A1}r_{A1}$-8	K-1	1455	-	NA[d]
$r_{A1}s_{A1}$-1	G-2	17035	135	No
			122	Yes (19-20)
$r_{A1}s_{A1}$-2[f]	H-1	13933	181	No
			36	Yes (20)
$r_{A1}s_{A1}$-3[f]	H-5	20567	390	No
			37	Yes (20)
$r_{A1}s_{A1}$-4[f]	H-5	13874	369	No
			75	Yes (20)
$r_{A1}s_{A1}$-5[f]	J-3	15951	367	No
			43	Yes (20)
$r_{A1}s_{A1}$-6	H-1	12786	395	No
			64	Yes (6)
$r_{A1}s_{A1}$-7	K-1	24276	500	Yes (6)

[a] Names for individuals consist of the genotype followed by a number designating the specific individual. Genotypes are based on allele-specific PCR ($s_{A1}s_{A1}$ for APHIS-S larvae and $r_{A1}r_{A1}$ or $r_{A1}s_{A1}$ for larvae from genetic linkage crosses).

[b] Samples are from the APHIS-S lab colony or specific F_1 backcross families.

[c] Numbers in parentheses indicate the exons affected for sequences with changes in the coding sequence (CDS).

[d] Not applicable, consensus was not obtained.

[e] Variants (Bt4-R2e-i) not previously identified by cloning and sequencing (see Table 1).

[f] Additional larvae found to have exon 6 cDNA deletion when LAA analysis included reads ≥3 kb.

Supplementary Table S4. Quantitative ELISA analysis for Bt proteins within Bollgard® II bolls of India field-grown cotton.

Boll/Larva Name[a]	District, State	[Cry1Ac] (µg per g tissue)[b]	[Cry2Ab] (µg per g tissue)[b]
AM-8	Ahmednagar, Maharashtra	1.60	19.6
CK-1	Chitradurga, Karnataka	1.41	22.3
GAP-2	Guntur, Andhra Pradesh	1.36	21.7
GAP-3	Guntur, Andhra Pradesh	1.06	20.6
JM-9	Jalna, Maharashtra	1.01	19.0
KT-1	Karimnagar, Telangana	1.08	17.6
KT-8	Karimnagar, Telangana	1.21	22.2
KT-10	Karimnagar, Telangana	1.14	18.4
RK-1	Raichur, Karnataka	1.27	18.3
RK-11	Raichur, Karnataka	1.65	20.6
YK-1	Yadgir, Karnataka	1.84	3.20

[a] Fourth instar pink bollworm larvae were collected in Bollgard II® cotton bolls from 7 districts and 4 states throughout south central India. Boll/larval samples are denoted by the first one-to-two letters of the district and the state it was collected from.

[b] ELISA plate assays were used to estimate the concentration of both Cry1Ac and Cry2Ab Bt toxin (micrograms per gram wet tissue) present in boll tissue for which pink bollworm larval survivors were recovered.

SUPPLEMENTARY FIGURE LEGENDS

Supplementary Figure S1. *PgABCA2* cDNA sequence. Nucleotide and deduced amino acid sequence of full-length *PgABCA2* cDNA. The predicted protein includes amino- and carboxyl-termini (pink), transmembrane regions TM1-TM12 (orange), intracellular loops ICL1-ICL5 (blue), extracellular loops ECL1-ECL6 (green), and nucleotide binding (Walker A, B and C) domains (purple). Putative exons 1-31 are numbered with grey tiles. Transmembrane spanning regions were predicted by Hidden Markov models (TMHMM) and displayed using Geneious 10.2.2.

Supplementary Figure S2. Sequence alignments of *PgABCA2* variants from APHIS-S, AZP-R, and Bt4-R2 strains. Consensus nucleotide sequence alignment of *PgABCA2* cDNA sequences from APHIS-S (identical to AZP-R) and Bt4-R2 strains reveals variation in Bt4-R2. cDNA variants Bt4-R2a-d were obtained from Bt4-R2 by cloning whereas Bt4-R2e-i were obtained by PacBio® sequencing.

Supplementary Figure S3. Partial genomic DNA sequencing of *PgABCA2* r_{A1} mutation site between APHIS-S and Bt4-R2. The r_{A1} mutation was obtained by comparing consensus sequences obtained from multiple cloned fragments corresponding to putative exon 18 to exon 21 within gDNA from APHIS-S and Bt4-R2. From Bt4-R2, a 44-bp deletion with 7-bp insertion is located at the 3'-end of exon 20 and spans the splice junction between exon 20 and intron 20. A 1,129-bp insertion within intron 18 is also unique to Bt4-R2. The insertion in intron 18 is shown as orange tile, the r_{A1} indel is shown as blue tile, and exons 18-21 and introns 18-20 are shown as gray and yellow tiles, respectively.

Supplementary Figure S4. Survival and weight of pink bollworm from 42 backcross (BC) families in the linkage experiment. Each BC family was produced by a single-pair cross between Bt4-R2 and either a male (blue bars) or female (pink bars) F_1 (Bt4-R2 X APHIS-S). (a) Survival (%). (b) Mean weight (plus standard deviation) of survivors.

Supplementary Figure S5. Partial genomic DNA sequencing of exons 5 to 9 for *PgABCA2* from APHIS-S and Bt4-R2. Genomic DNA corresponding to exons 5 to 9 of *PgABCA2* was PCR amplified, cloned and Sanger sequenced from APHIS-S or backcross survivors from the genetic linkage cross. Ten clones from each strain were aligned using MUSCLE alignment module within Geneious 10.1.2. No changes in gDNA corresponding to exon 6 or adjacent introns were found, indicating that variants with missing exon 6 are caused by mis-splicing. Analysis included 19 clones (14 from $r_{A1}s_{A1}$ *-3*, 3 from $r_{A1}s_{A1}$ *-4*, and 2 from $r_{A1}s_{A1}$ *-6*) obtained from individuals with the exon 6 cDNA deletion. A subset of ten clones is shown. Exons and introns are shown as gray and yellow tiles, respectively.

Supplementary Figure S6. Sequence alignments of *PgABCA2* partial genomic DNA and transcript variants. Genomic DNA corresponding to partial exons 19 to 21 of *PgABCA2* was PCR amplified, cloned and Sanger sequenced from pools of Bt4-R2 individuals. Three gDNA clones from Bt4-R2 were aligned with APHIS-S gDNA and transcript variants Bt4-R2a-i using MUSCLE alignment module within Geneious 10.1.2. Changes in gDNA corresponding exon 20 and adjacent introns were found and indicate the cause of mis-splicing of pre-mRNA that result in transcript variants. The r_{A1} indel in exon 20 and intron 20 is shown as blue tile. Exons and introns are shown as gray and yellow tiles, respectively.

Supplementary Figure S7. Sequence alignment of *PgABCA2* variants from Cry2Ab-susceptible and resistant pink bollworm from India. Multiple sequence alignment of *PgABCA2* consensus cDNA sequences from Cry2Ab-susceptible individuals from Akola, Maharashtra (AMH), India (note that AMH-1 is the representative consensus sequence generated from three individuals, AMH-1 to -3) and eight Cry2Ab-resistant individuals from Ahmednagar, Maharashtra (AM-8), Chitradurga, Karnataka (CK-1), Guntur, Andhra Pradesh (GAP-3), Karimnagar, Telangana (KT-1, -8, and 10), and Raichur, Karnataka (RK-1 and -11) was performed using MUSCLE within Geneious 10.1.2. The full-length coding sequence for *PgABCA2* from the U.S. Cry2Ab-susceptible strain (APHIS-S) was included as reference and corresponding exons 1-31 are shown as gray tiles.

Supplementary Figure S8. Sequence alignments of *PgABCA2* partial genomic DNA from Cry2Ab-resistant pink bollworm collected from Chitradurga, Karnataka (CK-1), India. Nucleotide alignment of *PgABCA2* gDNA fragments (five clones) corresponding to exon 3 to exon 7 from a Cry2Ab-resistant pink bollworm collected from Bt cotton in Chitradurga, Karnataka (CK-1), India. The four known cDNA variants obtained from this individual (CK-1a-d) and the corresponding *PgABCA2* gDNA sequence from the U.S. Cry2Ab-susceptible strain (APHIS-S) were included for reference. Sequences were aligned using MUSCLE within Geneious 10.1.2. Exons and introns are shown as gray and yellow tiles, respectively. The insertion in exon 4 is shown as blue tile. The location of alternative 5' and 3' splice sites are indicated by orange and red arrows, respectively.

Supplementary Figure S9. Sequence alignments of *PgABCA2* partial genomic DNA from Cry2Ab-resistant pink bollworm collected from Guntur, Andhra Pradesh (GAP-3), India. Nucleotide alignment of *PgABCA2* gDNA fragments (ten clones) corresponding to exon 4 to exon 9 from a Cry2Ab-resistant pink bollworm collected from Bt cotton in Guntur, Andhra Pradesh (GAP-3), India. The two known cDNA variants obtained from this individual (GAP-3a and GAP-3b) and the corresponding *PgABCA2* gDNA sequence from the U.S. Cry2Ab-susceptible strain (APHIS-S) were included for reference. Sequences were aligned using MUSCLE within Geneious 10.1.2. Exons and introns are shown as gray and yellow tiles, respectively. The single-nucleotide mutation within the exon-intron 8 splice site is marked by a green tile, with the corresponding alternative 5' site indicated by an orange arrow.

Supplementary Figure S10. Sequence alignments of *PgABCA2* partial genomic DNA from Cry2Ab-resistant pink bollworm collected from Karimnagar, Telangana (KT-1), India. Nucleotide alignment of *PgABCA2* gDNA fragments (two clones) corresponding to exon 5 to exon 7 from a Cry2Ab-resistant pink bollworm collected from Bt cotton in Karimnagar, Telangana (KT-1), India. The three known cDNA variants obtained from this individual (KT-1a-c) and the corresponding *PgABCA2* gDNA sequence from the U.S. Cry2Ab-susceptible strain (APHIS-S) were

included for reference. Sequences were aligned using MUSCLE within Geneious 10.1.2. Exons and introns are shown as gray and yellow tiles, respectively.

Supplementary Figure S11. Sequence alignments of *PgABCA2* partial genomic DNA from Cry2Ab-resistant pink bollworm collected from Raichur, Karnataka (RK-1), India. Nucleotide alignment of *PgABCA2* gDNA fragments (9 clones) corresponding to exon 1 to exon 11 from a Cry2Ab-resistant pink bollworm collected from Bt cotton in Raichur, Karnataka (RK-1), India. The three known cDNA variants (RK-1a-c) corresponding to this gDNA region and the corresponding *PgABCA2* gDNA sequence from the U.S. Cry2Ab-susceptible strain (APHIS-S) were included for reference. Sequences were aligned using MUSCLE within Geneious 10.1.2. Exons and introns are shown as gray and yellow tiles, respectively. The insertion in exon 4 is shown as blue tile. The location of alternative 5' and 3' splice sites are indicated by orange and red arrows, respectively.

Supplementary Figure S12. Sequence alignments of *PgABCA2* partial genomic DNA from Cry2Ab-resistant pink bollworm collected from Ahmednagar, Maharashtra (AM-8), India. Nucleotide alignment of *PgABCA2* gDNA fragments (five clones) corresponding to exon 17 to exon 18 from a Cry2Ab-resistant pink bollworm collected from Bt cotton in Ahmednagar, Maharashtra (AM-8), India. *PgABCA2* gDNA sequence from the U.S. Cry2Ab-susceptible strain (APHIS-S) was included for reference. Sequences were aligned using MUSCLE within Geneious 10.1.2. Exons and introns are shown as gray and yellow tiles, respectively. The 5-bp deletion in exon 17 is shown as blue tile.

Supplementary Figure S13. Unprocessed agarose gel showing r_{A1} allele-specific PCR. PCR using the allele-specific primers rA1-F and rA1-R (corresponding to Fig. 2B) yielded a 343-bp fragment from APHIS-S ($s_{A1}s_{A1}$) (Lane 1), a 305-bp fragment from Bt4-R2 and F_2 $r_{A1}r_{A1}$ offspring (Lanes 2, 5-6), and both fragments in the offspring from crosses between the two strains ($r_{A1}s_{A1}$) (Lanes 3-4). Lane M corresponds to 1kb Plus DNA Ladder (Life Technologies).

Supplementary Figure S1

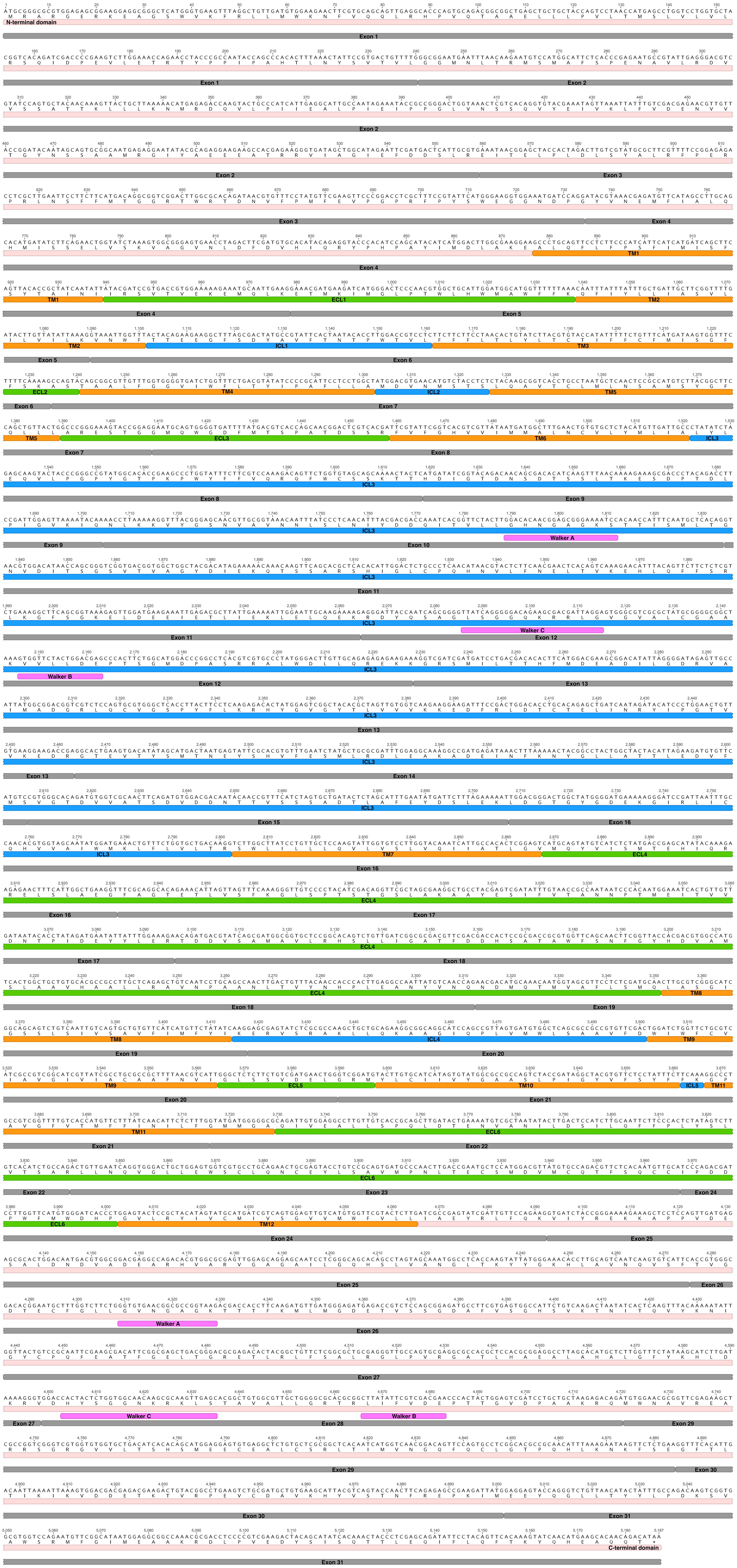

Supplementary Figure S2

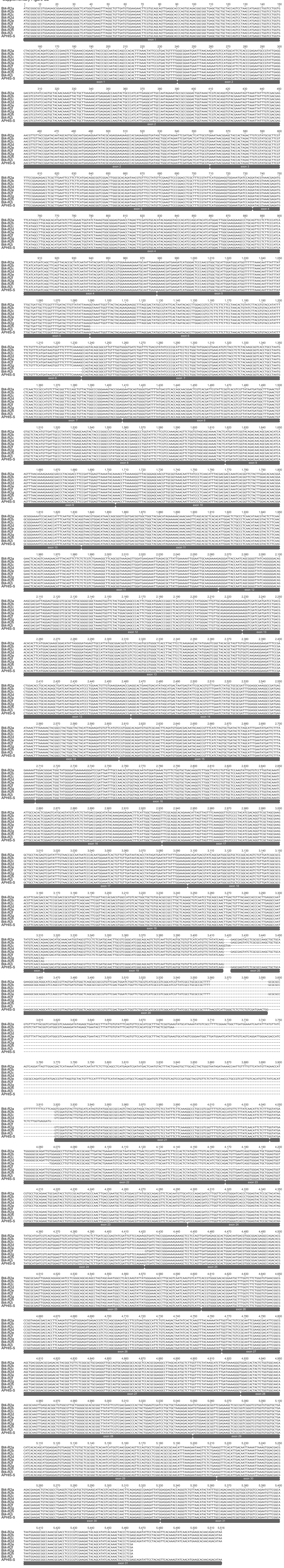

Supplementary Figure S3

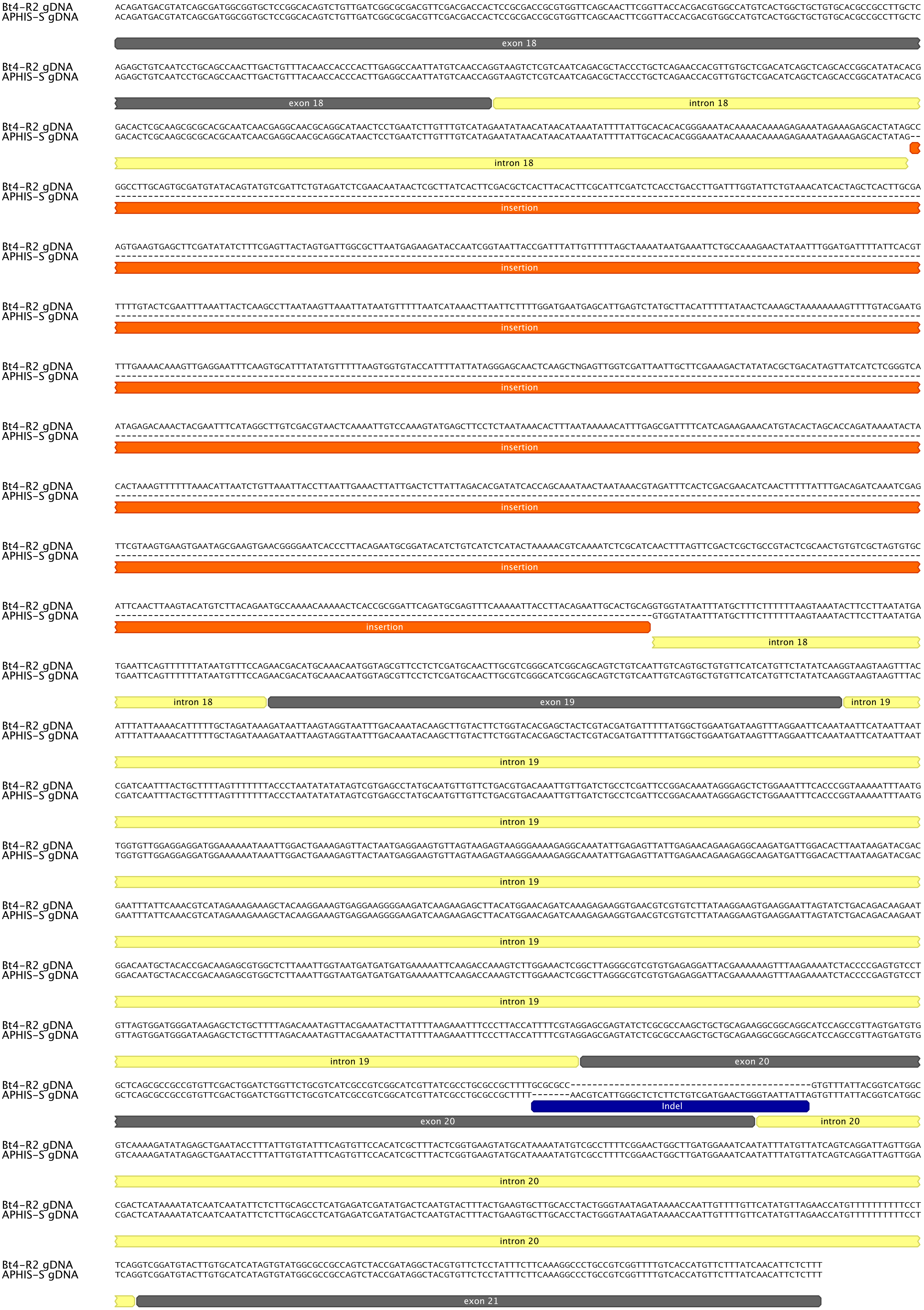

Supplementary Figure S4

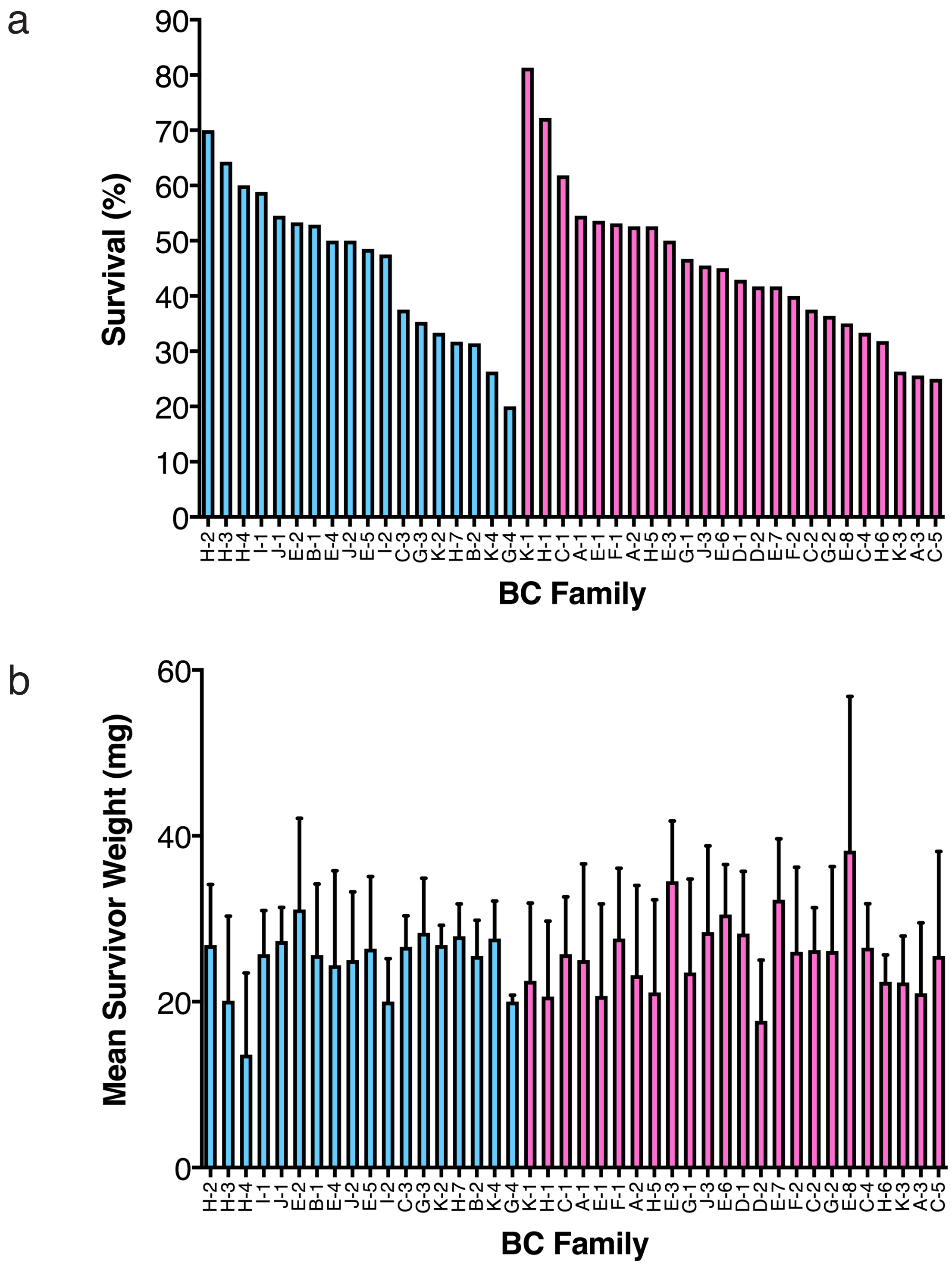

Supplementary Figure S5

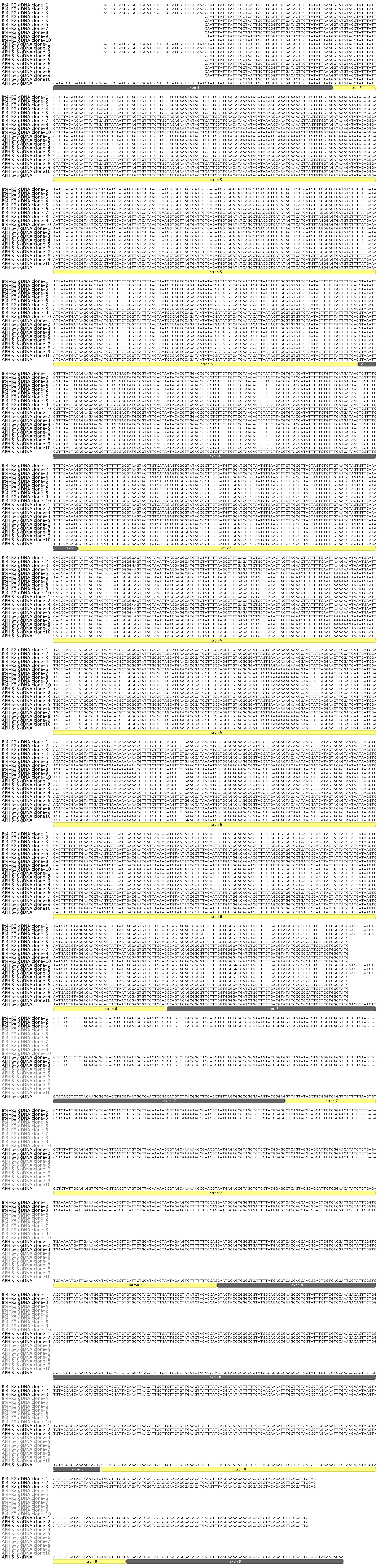

Supplementary Figure S6

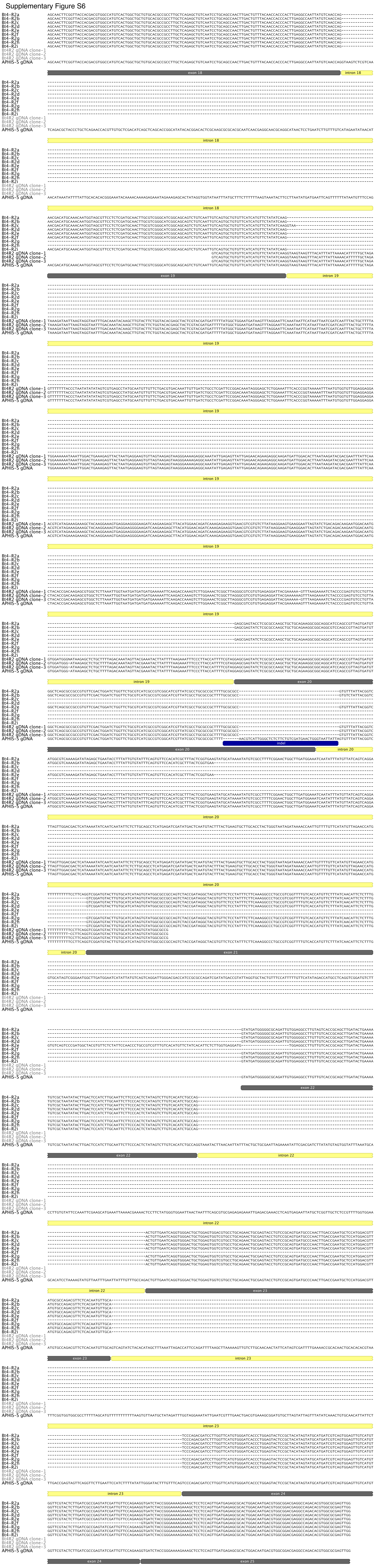

Supplementary Figure S7

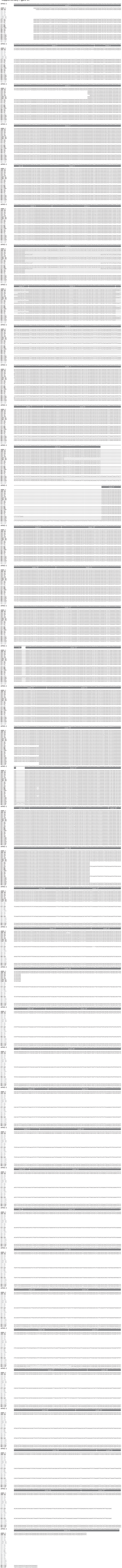

Supplementary Figure S8

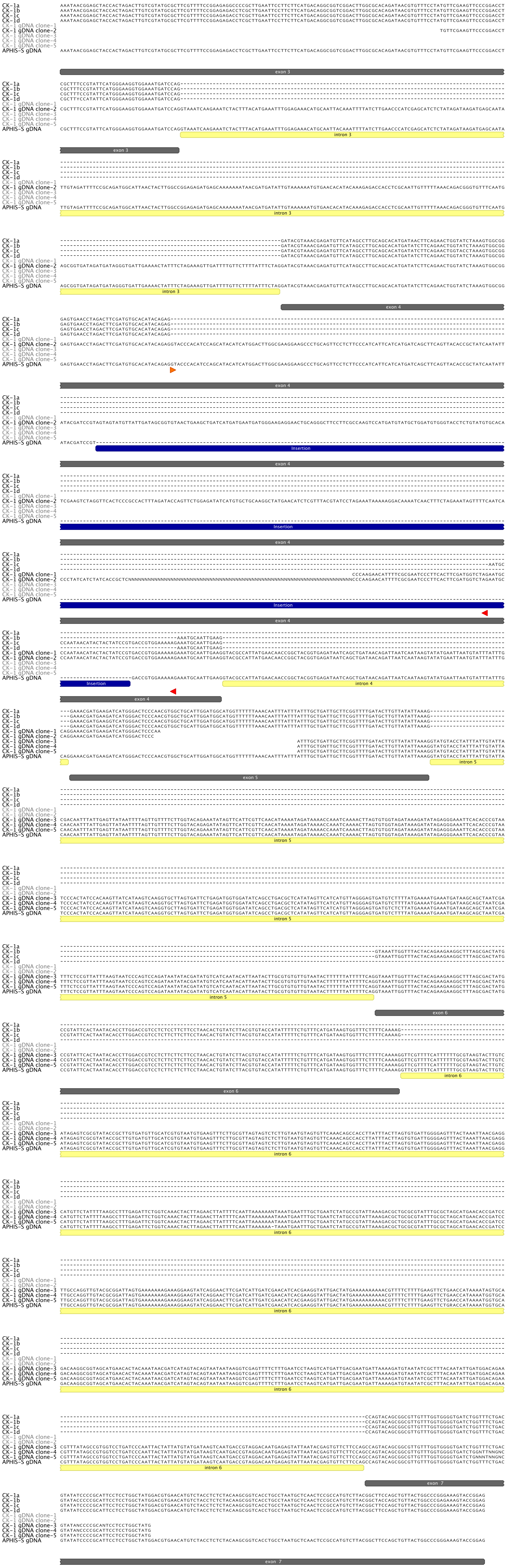

Supplementary Figure S9

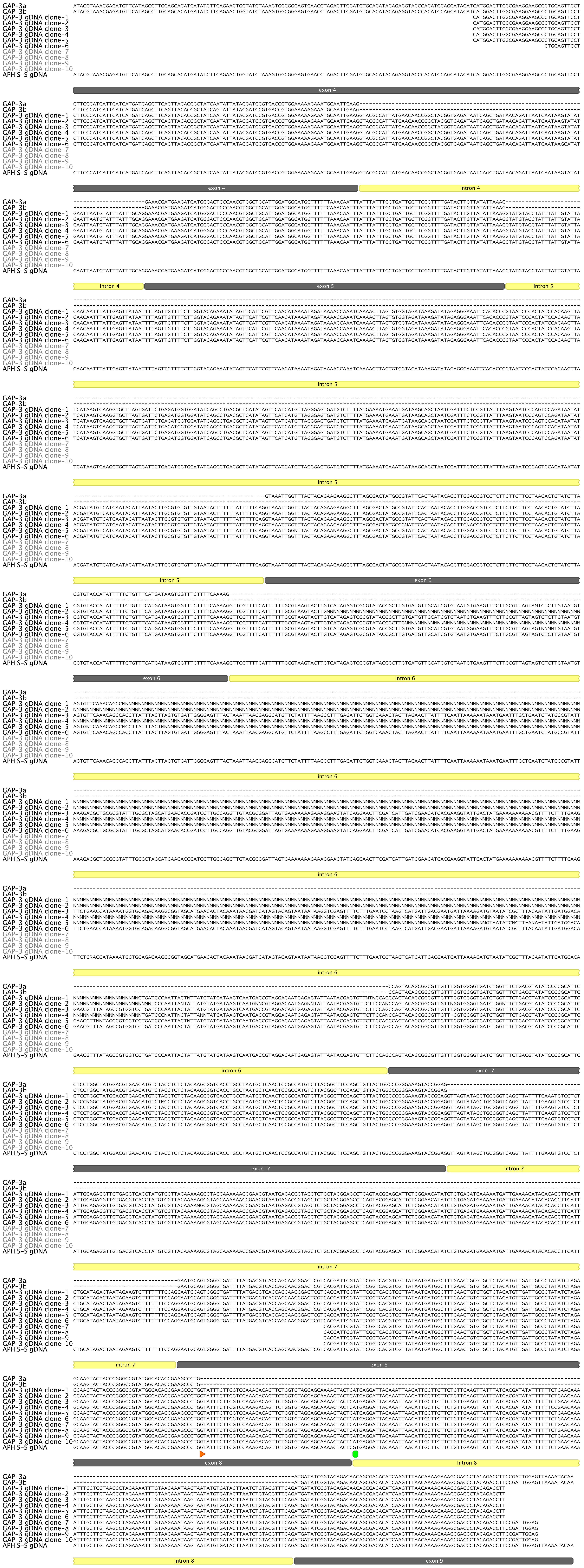

Supplementary Figure S10

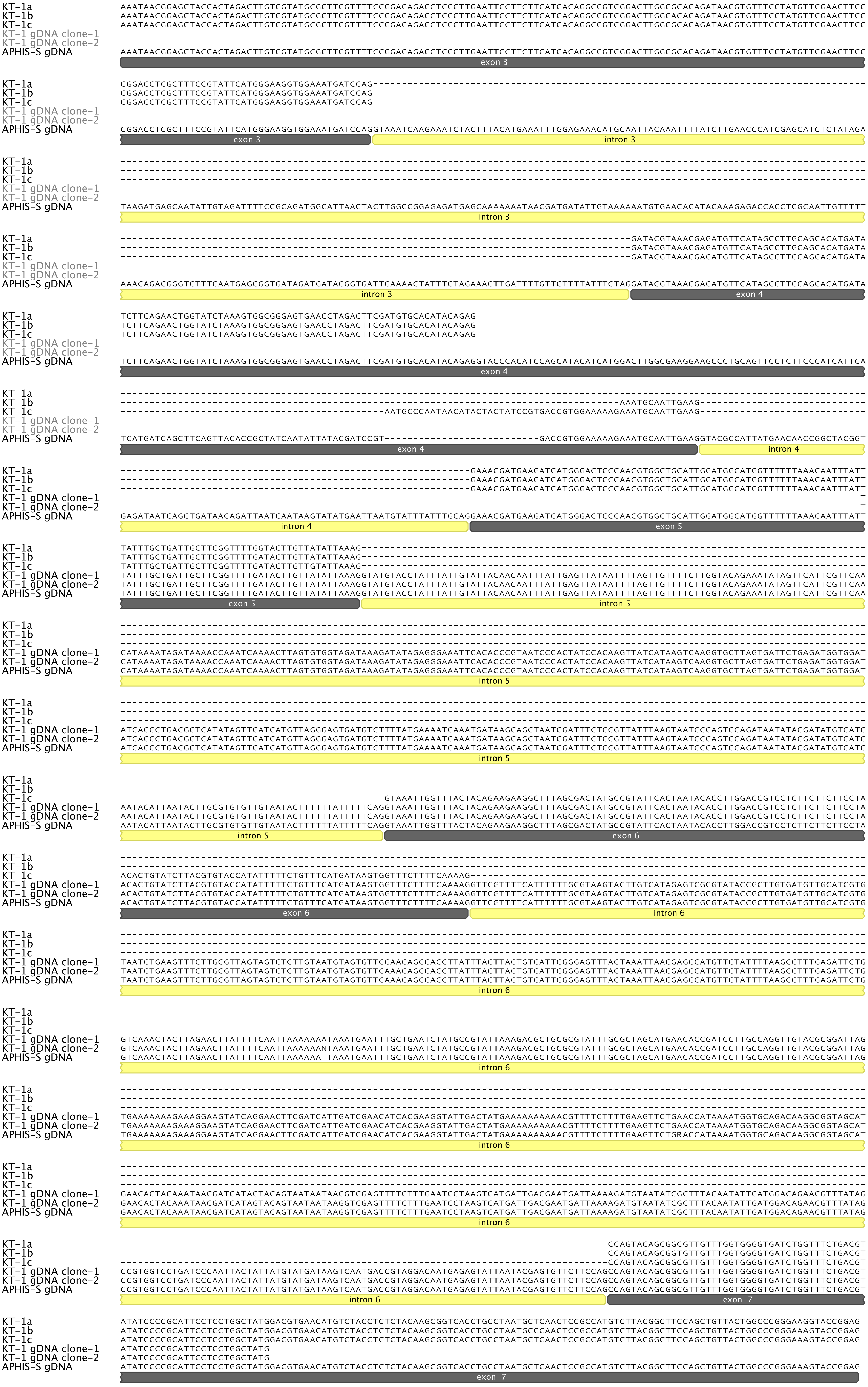

Supplementary Figure S11

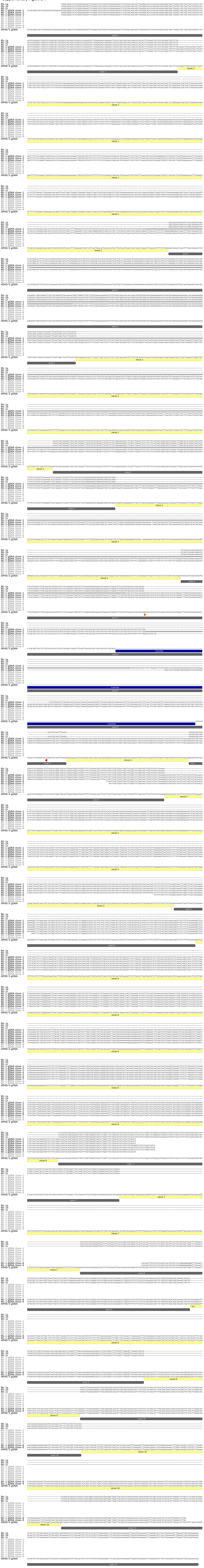

Supplementary Figure S12

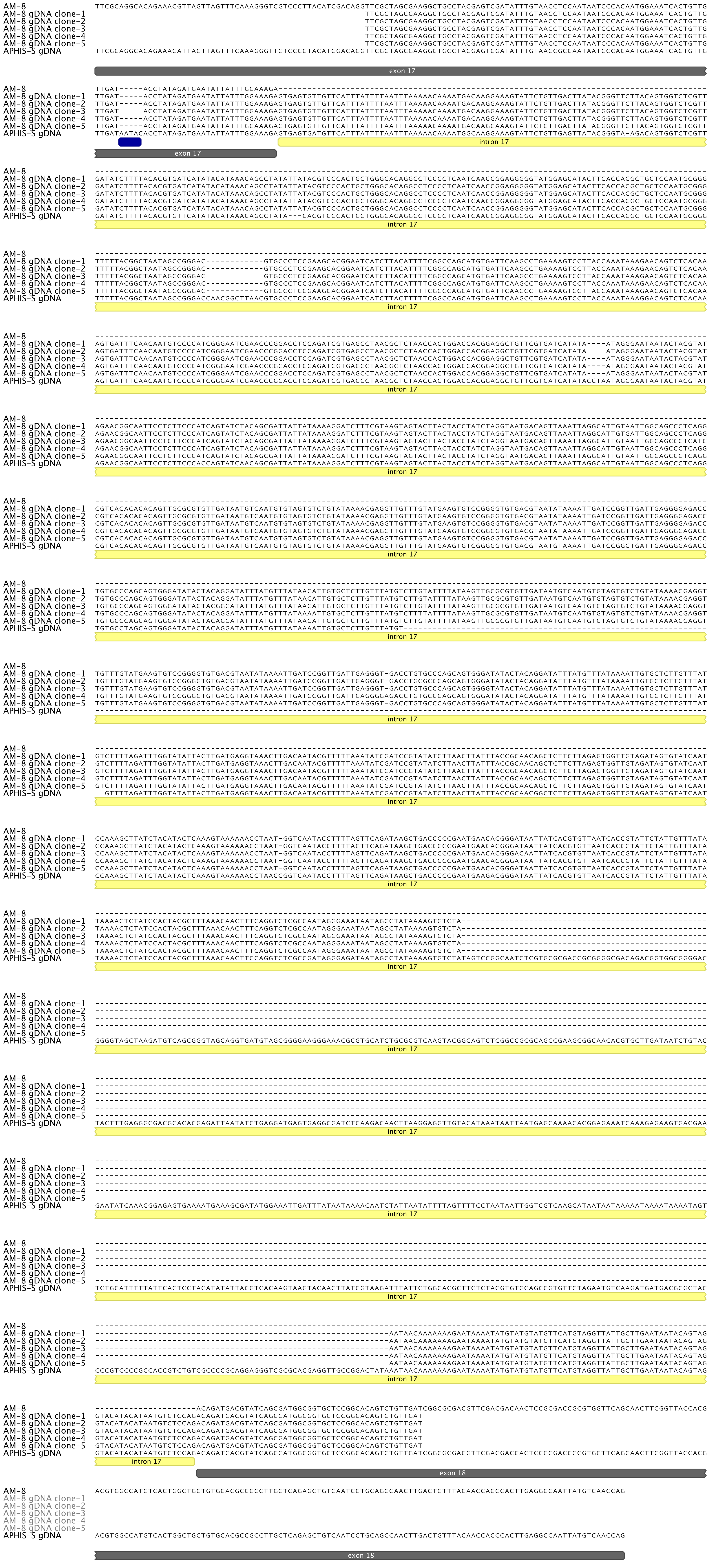

Supplementary Figure S13

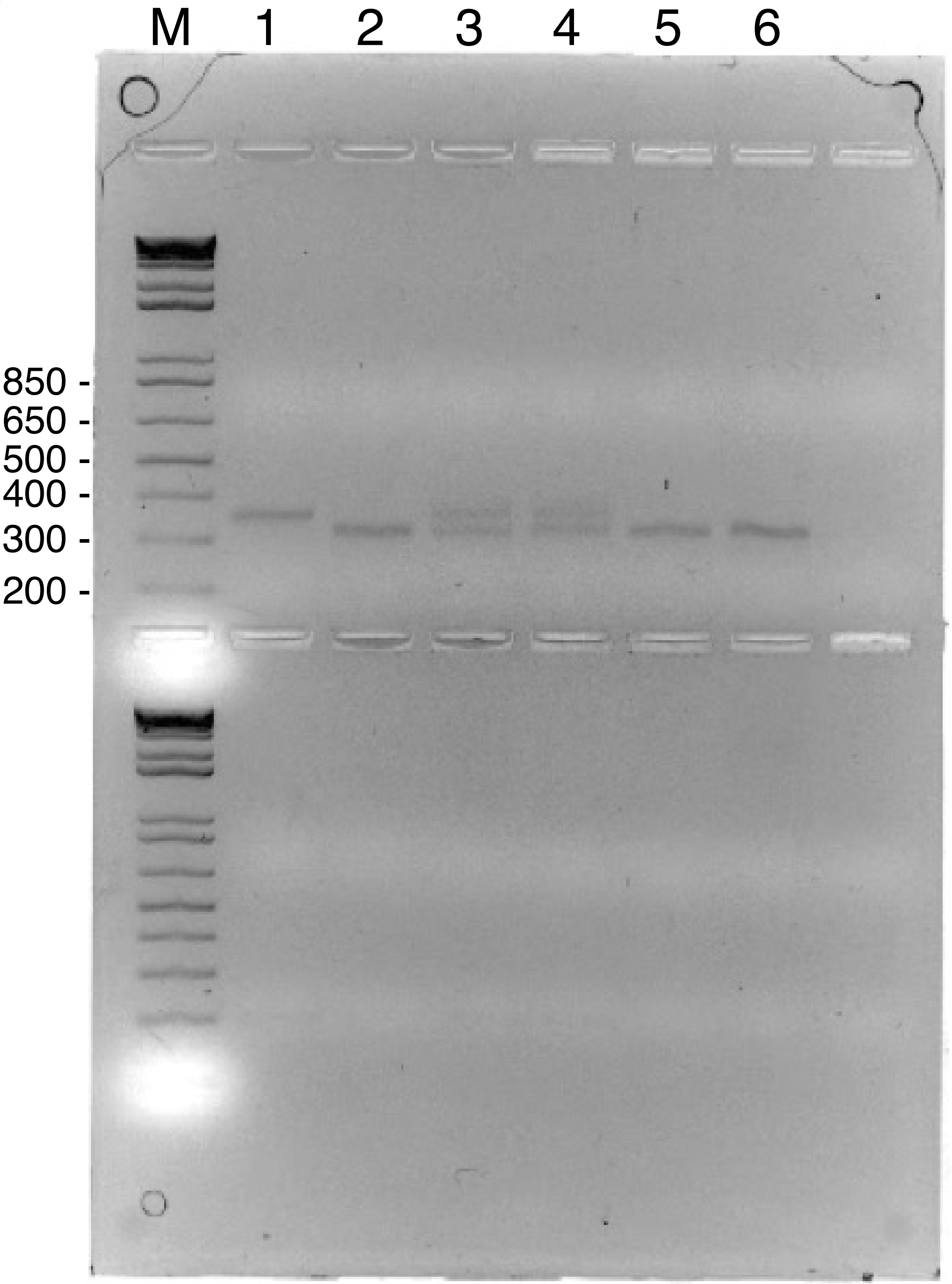

Supplement: Supplementary file 1 — Supplementary Information [file 41598_2018_31840_MOESM1_ESM.pdf]
